# Recent Trends in Upconversion Luminescent Inorganic Materials and Nanomaterials for Enhanced Photovoltaic Solar Cell and Biological Applications

**DOI:** 10.1007/s10895-025-04198-x

**Published:** 2025-03-10

**Authors:** Mostafa S. Eraky, Sara S. Elsherif, Moustafa M. S. Sanad

**Affiliations:** Central Metallurgical Research & Development Institute, P.O. Box: 87, Cairo, 11421 Helwan Egypt

**Keywords:** Luminescent materials, Upconversion, Synthesis, Solar cell, Biological

## Abstract

Upconversion (UC) luminescent materials have emerged as captivating contenders in revolutionizing both photovoltaic (PV) solar cell efficiency and biological capabilities. Their unique ability to convert low-energy infrared light into high-energy visible or ultraviolet (UV) photons unlocks untapped resources in the solar spectrum and allows for deeper tissue penetration in biological imaging. By bridging the gap between recent advancements and remaining hurdles, we aim to inspire further research and accelerate the translation of these materials into practical and impactful applications for both energy and healthcare. This review delves into the recent trends propelling these materials forward. We explore advancements in UC efficiency through optimized material design, novel synthesis routes, and synergistic integration with existing technologies. In the domain of PVs, we shed light on strategies utilizing UC to address spectral mismatch and enhance light harvesting, paving the way for higher power conversion efficiencies. For biological applications, we illuminate the development of biocompatible and targeted UC probes, enabling deep tissue penetration, multimodality imaging, and theranostic potential. We critically analyze the current limitations and future directions of these materials, highlighting the challenges of toxicity, quenching, and scalability that remain to be tackled. By providing a comprehensive overview of the exciting progress and persistent hurdles in UC research, this review aims to guide future explorations and catalyze the widespread adoption of these materials in sustainable energy generation and advanced medical diagnostics.

## Introduction

**Fig. 1:**
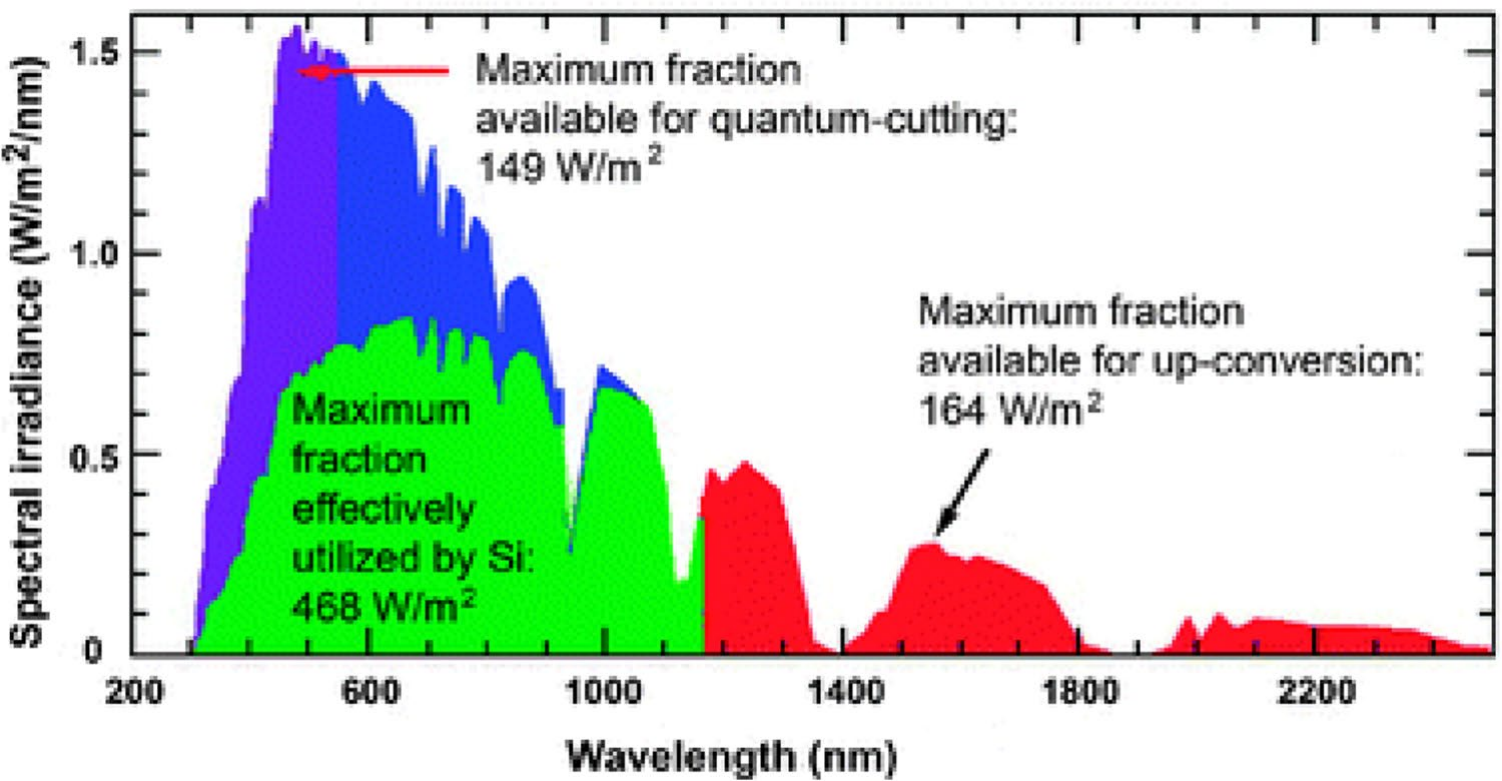
Solar spectrum at AM 1.5G illustrating the working range of commercial Si solar cell, up and down conversions possible working ranges [4]

The efficient use of solar energy for photovoltaics (PVs) is currently still a major challenge because the current solar cells only use a relatively small part of the incident sunlight. The reason for this phenomenon is that only photons with an energy higher than the bandgap of active materials can be absorbed, while the photons below the bandgap are completely wasted [1]. Converting the incident sunlight below the bandgap into the light above the bandgap using a UC layer has been shown to be an effective method to significantly reduce energy losses. Recently, it was reported that the limiting efficiency of a solar cell in combination with a boost converter layer would exceed the Shockley-Queisser limit [2]. Moreover, the thermal loss of the low-energy part of the light spectrum is one of the most known reasons for solar cell shortage. Accordingly, UC materials utilize this lost energy through a process called mixed thermos-photovoltaic (TPV), which contributes 73% as a predicted maximum conversion efficiency [3]. Using such an approach is very exciting because it is easily applicable to all the traditional existing solar cells in the market through some modifications. In addition, both parts solar cells and the UC layer could be modified and processed separately [4]. Besides, upconversion nanophosphors were developed to be applied in other fields including photocatalysis, biomedical, biosensing, bioimaging, optogenetics, drug release, and photodynamic therapy [5–9]. The ideal upconverter material should possess different features to be effective for these various applications. Firstly, the UC candidate should have a broadband excitation range covering the absorption range of the solar cell's active material. Secondly, the upconverter emission range should be shorter than the absorption range of the active material. Thirdly, UC material should display a good response for low energy photons, and it should have a high UC capability [1]. In addition to these technical features, UC materials should be stable, non-toxic, environmentally friendly, and low-cost compared with noble metals. The potential working range of UC materials in the solar spectrum is illustrated in Fig. 1 [4].

The upconverter candidate consists of an inorganic host material, a sensitizer, and active ions. Each part has its own task. The host material is an essential part of the guest–host structure of the upconverter. It serves as a host field for the other parts of the upconverter and the entire UC process. Accordingly, it should be transparent, have a large optical damage edge, and be chemically stable [9, 10]. The photosensitizer acts as a light absorber in a low-energy photons region and transfers the absorbed photons to the activator component to be merged and emitted as a higher-energy photons region suitable for the absorption range of the target solar cell [9]. This review discusses the fundamentals and mechanisms of UC processes. Besides, the structure, properties, and preparation methods of upconverter materials will be discussed. The review study will cover the UC applications by providing examples of each application and their important reported results. Moreover, the most effective strategies for improving the performance of UC materials will be collected and comprehensively discussed. The review is closed with a comprehensive summary and conclusion about the most important collected points offering a futuristic view about this field of research.

## Fundamentals of Upconversion Luminescence

### History and Fundamentals

A process known as UC is the optical transformation of lower-energy photons into higher-energy photons. It has been extensively studied since the mid-1960s and is widely applied in optical devices. However, photon UC was primarily studied in bulk glasses or crystalline materials until the beginning of the twenty-first century, and much of its potential was unrealized. When UC nanomaterials were developed, the situation drastically changed. At the nanoscale level, numerous new effects start to operate, creating fresh opportunities for UC [11]. With the rapid advancement of nanotechnology over the past ten years, high-quality rare earth-doped UC nanoparticles have been successfully synthesized and increasingly used in biological sciences. Phase-based procedures like thermal breakdown, hydrothermal reaction, and ionic liquid-based synthesis are typically used in synthesis. The ability of UC nanoparticles to emit visible light when exposed to near-infrared (NIR) radiation sets them apart from other nanomaterials. In biological samples, NIR irradiation results in reduced auto-fluorescence reduced scattering and absorption, and deep penetration [12]. The rare earth (RE) elements, which include the lanthanide (Ln) series, yttrium, and scandium, are the main elements where the UC process has been observed. Ln^3+^ ions have particular 4fn 5d0–1 inner shell configurations that are well-shielded by outer shells with abundant and unique energy-level structures. Via intra-4f or 4f-5d transitions, these Ln^3+^ ions emit bright light. Their distinct luminescence characteristics, like their anti-Stokes emission, long-duration emission, and restricted bandwidth, have found extensive applications in solar cells, lasers, optical imaging, photodynamic treatment, and other fields. The trivalent rare earth element (RE^3+^) doped materials have multiple long-lived excited states and can exhibit highly efficient energy transfer processes that are responsible for UC emission under certain conditions, so they are among the most promising materials for energy UC [13].

Because most fluorescent materials still have problems when exposed to UV or visible light, scientists have been working to create new, well-shaped, high-quality nanoparticles called UC nanomaterials. Biological samples may experience auto-fluorescence and photodamage upon stimulation by UV or visible radiation, which would reduce the signal-to-noise ratio and sensitivity. Conversely, UC nanomaterials typically have an inorganic host doped with Ln^3+^ ions, which exhibit low cytotoxicity and good biocompatibility to a variety of cell lines [14, 15]. Presently, one of the primary limiting aspects is the low luminescence efficiency. Selecting a suitable host material with reduced phonon energy is crucial to achieving the maximum UC luminescence efficiency (high phonon frequencies of the host lattice contribute to non-radiative relaxation). Thus far, it has been established that certain host materials, such as fluoride, chloride, and bromide, can increase the intensity of UV light. Since most bromides and chlorides are moisture-sensitive, they are not appropriate for labeling biomolecules (which are primarily employed in aqueous solutions). [16]. They have been regarded as an ideal host material because of the high refractive index and great transparency resulting from low-energy phonons of the RE fluorides, primarily REF_3_ and AREF_4_ (A = alkali). These two benefits also result in a higher luminescence quantum yield and a decreased likelihood of non-radiative decay. Many lanthanide ions have the potential to go through the Near IR to visible UC process in theory. Meanwhile, at low pump densities (980 nm excitation), only a small number of trivalent lanthanide ions, such as Er^3+^ and Tm^3+^, may perform as highly effective UC. Yb^3+^, Er^3+^, Yb^3+^, or Tm^3+^ co-doped NaYF_4_ nanomaterials (NaYF_4_:Yb^3+^, Er^3+^ or NaYF_4_: Yb3^+^, Tm^3+^) are the most widely employed UC chemicals. One of the most UC efficient phosphors, for instance, Yb^3+^/Er^3+^ pair doped NaGdF_4_ has been studied. When excited to 976/980 nm infrared, the Yb^3+^/Er^3+^ combination produces bright green emission that is extensively researched for imaging in the green area. More specifically, UC luminescence emission was greater in the β-NaGdF_4_ phase (hexagonal) than in the α-phase (cubic) [17].

### Upconversion Mechanisms

Normally, UV and visible emissions can be produced from NIR excitation using UC nanomaterials doped with lanthanides. They demonstrated potential usefulness in a variety of applications such as photoluminescence bioimaging, PV energy conversion, lasers, displays, and optical refrigeration of solids because of their relatively high emission efficiency, low auto-fluorescence, excellent chemical and thermal photo-stability, deep tissue penetration, exceptional biocompatibility, low toxicity, color purity, and ease of surface functionalization. [18]. In principle, for enhancing solar cell efficiency, the ideal upconverter must have a broadband excitation range longer than the maximum absorption wavelength of active materials. In addition, the emission range of the upconverter is usually shorter than the energy gaps of active materials and is located in the spectra regions where solar cells have their maximum spectral response. Therefore, the upconverter always shows a good response under low-power excitation (in the range of 10–100 Wm^2^), achieving high UC efficiency [1].

There are five different mechanisms that regulate the UC: (i) excited state absorption (ESA) upconversion, (ii) energy transfer upconversion (ETUC) and cooperative upconversion (CUC), (iii) photon avalanche (PA) upconversion, (iv) Triplet–triplet annihilation upconversion (TTA-UC), (v) energy migration-mediated upconversion (EMUC) [19, 20].

#### Excited State Absorption Upconversion

**Fig. 2:**
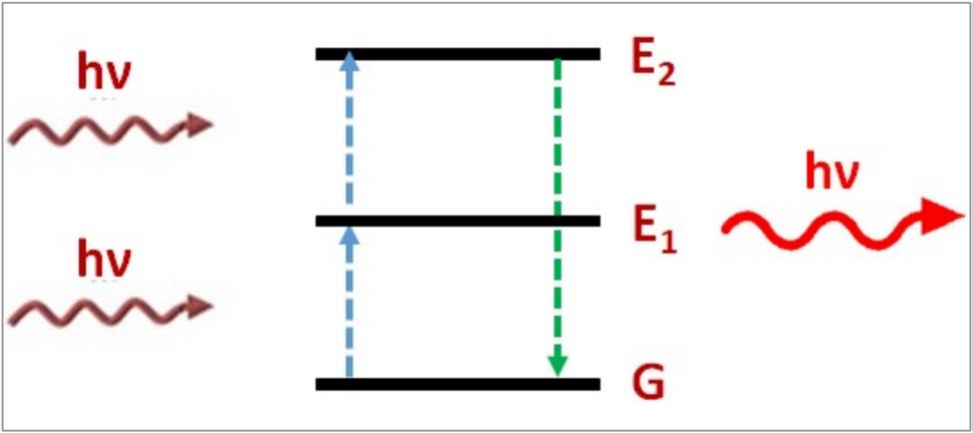
Excited state absorption upconversion [19]

Using a hypothetical three-level system, as illustrated in Fig. 2, is a straightforward technique for excited state absorption (ESA) upconversion. When the first photon is absorbed, the emission center moves from its original ground state (1) to the intermediate excited state (2). The center is promoted to the higher-lying excited state (3) if it absorbs a second photon before relaxing back to the ground state. A photon with an energy twice that of the two absorbed photons is subsequently emitted when the center radiatively relaxes back to state 1. State 2 must be long enough and have a strong enough photon flux so that a second photon is absorbed before state 2 relaxes back to its initial state for ESA to occur [21].

#### Energy Transfer Upconversion

**Fig. 3:**
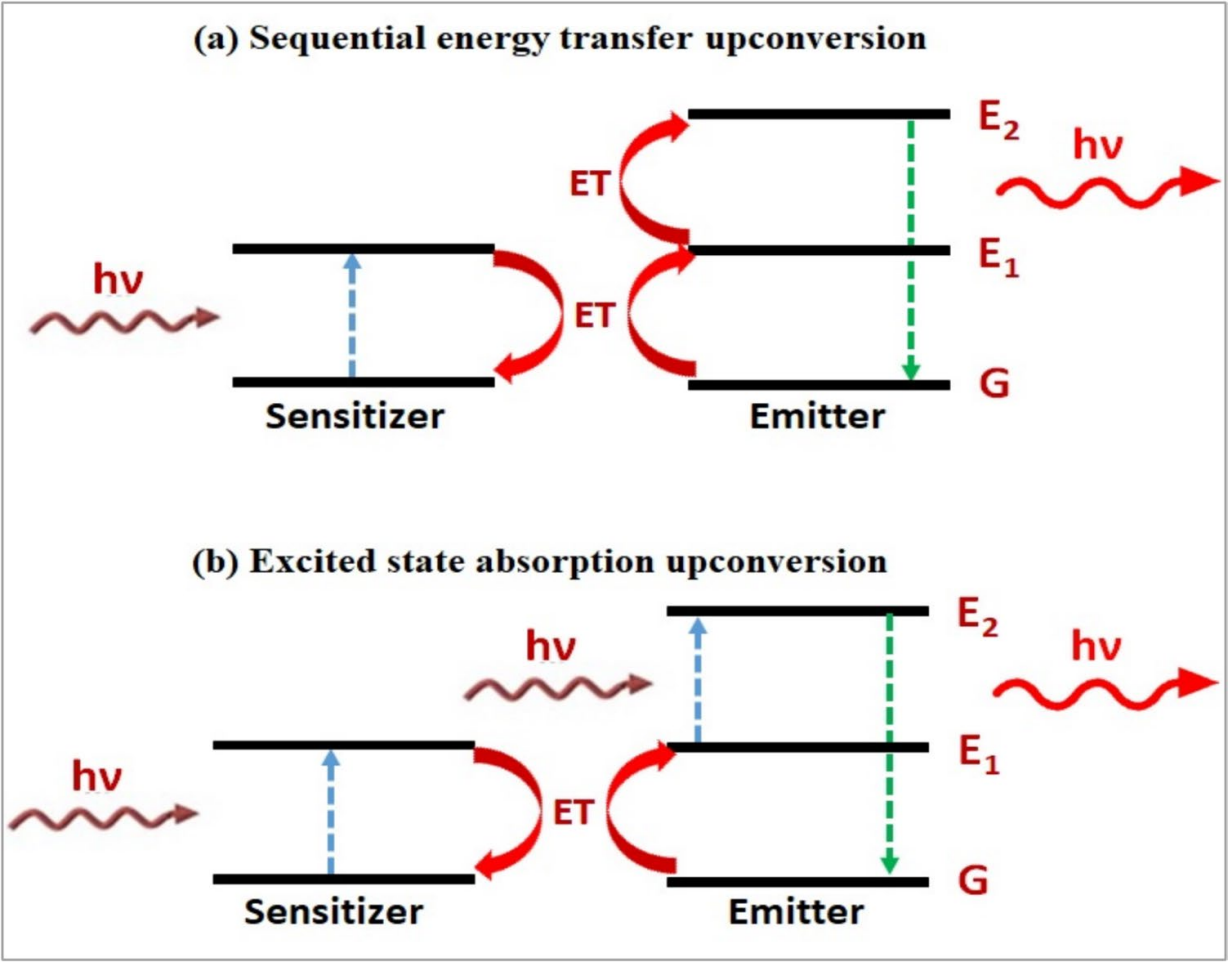
Sequential energy transfer upconversion (left) and energy transfer followed by excited state absorption upconversion (right) [19]

Energy transfer upconversion (ETUC) produces the UC by use of a sensitizer and emitter, which are usually two distinct kinds of rare-earth ions. On the left side of Fig. 3, you can see the most basic ETUC mechanism. A photon is firstly absorbed by a sensitizer, which is then activated. Energy transfer (ET) to the emitter, which raises it to the intermediate excited state (2), and sensitizer relaxation back to the ground state occurs next. The emitter is then promoted to the higher-lying excited state (3) by ET to the second sensitizer, which absorbs a second photon. From there, it radiatively relaxes back to the ground state, emitting a higher energy photon. As demonstrated, ET can also happen in tandem with other procedures like ESA. The sensitizer and emitter need to be geographically close for ET to occur, and the energy of the emitter's intermediate excited state needs to be lower than the sensitizer's excited state in order to provide an energetic driving force for an efficient ETUC [12, 22].

#### Photon Avalanche (PA) Upconversion

**Fig. 4:**
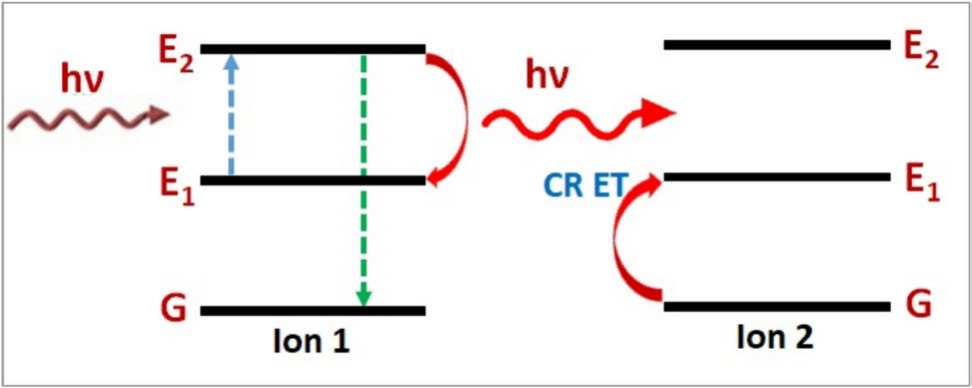
Photon avalanche upconversion [21]

Within laser cavities, there is an uncommon phenomenon called photon avalanche (PA) upconversion. As seen in Fig. 4, the PA mechanism is based on cross-relaxation energy transfer (CR ET) between ions that are closely separated within a material. Every ion is in state 1 at first, but eventually, one will be promoted to state 2, and ultimately to state 3 through ESA. This ion can then promote a nearby ion to state 2 and relax back to state 2 through the CRET mechanism. After that, these two ions can go through CR ET and ESA with two additional nearby ions, making four ions in state 2, and so on, until all the ions in the material are in state 2. After that, UC moves straight from state 2 to state 3 through excited state absorption without first undergoing ground state absorption [21, 23].

#### Triplet–triplet Annihilation Upconversion

**Fig. 5:**
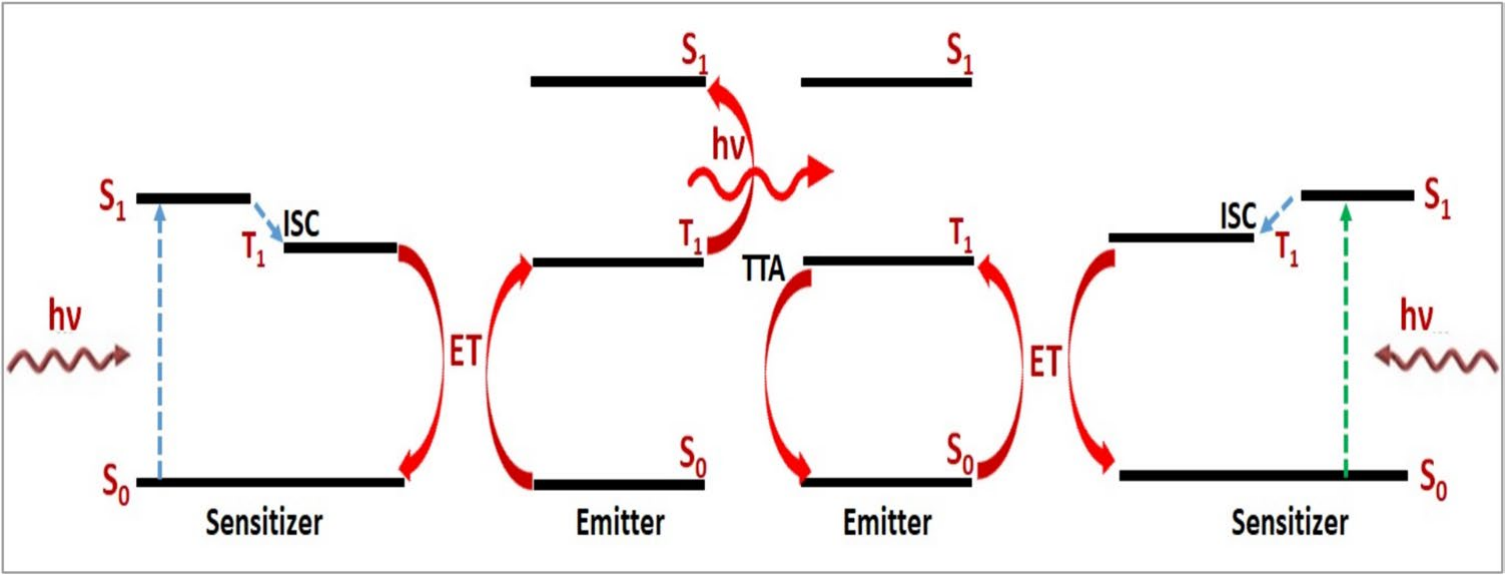
Schematic of sensitized triplet–triplet annihilation upconversion [20]

The fourth type of UC mechanism is called triplet–triplet annihilation (TTA), which inherently includes a variation of ETUC in molecular systems due to the presence of localized excited states, resulting in distinct singlet and triplet states as illustrated in Fig. 5. Sensitized triplet–triplet annihilation (sTTA) facilitates the transformation of low-energy photons into high-energy ones and has been suggested as an effective approach for managing non-coherent photons in multicomponent systems. This process harnesses the interaction between two optically dark triplet states of emitter molecules to generate higher-energy photons [24]. More precisely, TTA is a bimolecular process in which photons are absorbed by a sensitizer molecule and unconverted photons are released by an emitter molecule. A photon is initially promoted to its excited singlet state (S_1_) by a sensitizer, and then it undergoes intersystem crossing (ISC) to reach the triplet state (T_1_). The emitter is subsequently promoted to the T_1_ by means of triplet-mediated ET, which transfers the energy to the emitter. For a second pair of emitter-sensitizers, repeat this operation. Ultimately, TTA is applied to both emitters, elevating one to the S_1_ and the other to the ground state. After radiatively relaxing to the ground state, the emitter in the S_1_ releases a high-energy photon [25]. Therefore, TTA was reported to be a UC mechanism for reducing thermal and spectral losses, hence increasing the power conversion efficiency in PV solar cells and optoelectronic devices [25].

Like ETUC, Sensitizers and activators are also used in the cooperative UC process; the activator in this process differs only in that it lacks an intermediate energy level. At EMUC, there are four different kinds of luminous centers: sensitizer, accumulator, migrator, and activator. In a traditional EMUC mechanism, energy is transferred to the migrator through the core–shell contact after the ETUC process fills the accumulator to a higher excited state. This is accompanied by randomized energy leaping into the migrator ions. After that, the activator traps the migrating energy. ETUC is well-liked in comparison to other methods because of its high upconversion efficiency, which necessitates that each of the two adjacent ions pump an identical energy photon. Sensitizers and activators are also used in the cooperative UC process; the activator in this process differs only in that it lacks an intermediate energy level. At EMUC, there are four different kinds of luminous centers: sensitizer, accumulator, migrator, and activator. In a traditional EMUC mechanism, energy is transferred to the migrator through the core–shell contact after the ETUC process fills the accumulator to a higher excited state. This is accompanied by randomized energy leaping into the migrator ions. After that, the activator traps the migrating energy. ETUC is well-liked in comparison to other methods because of its high UC efficiency, which necessitates that each of the two adjacent ions pumps an identical energy photon [26]. TTA allows unique properties such as tunable spectral range, enhanced UC efficiency at non-coherent solar spectrum, large absorption efficiency, low excitation power density, and high quantum yield [27, 28]. These properties make the research consider the mechanism of TTA for the manufacturing of high-performance blue organic light-emitting devices (OLEDs) [29]. On the other hand, low-power TTA was utilized to produce a red-light tunable soft controller with a high quantum yield of up to 9.3% [30]. This consequently provides a new technological application of the TTA-based UC system in photonic devices. Other recent studies in 2023 have figured out TTA as an effective way for high safety, low probability of photodamage, high quantum yield, low excitation power density, and tunable absorption and emission wavelengths in bioimaging applications [31]. Further, upgraded TTA-UC materials were developed and applied as promising candidates for invivo bioimaging in whole animal deep imaging with high accuracy by controlling the singlet oxygen generation [32]. It is also applied in bioimaging through deep tissue cancer cells that operate in the red or NIR excitation wavelengths with great properties of non-toxicity, and biocompatibility [33].

## Synthesis and Characterization of Upconversion Materials

### Synthesis of Upconversion Materials

UC materials, particularly lanthanide-doped nanocrystals (NCs), have garnered significant attention due to their unique ability to convert low-energy photons (such as NIR) into high-energy photons (visible or UV). This property makes them promising candidates for a variety of applications, including PVs, bioimaging, and anti-counterfeiting. Designing upconversion luminescent materials involves several criteria to achieve efficient photon upconversion, which is the process of converting lower-energy photons into higher-energy ones. Here are some key considerations:

#### Energy Levels of Excited States

The material should possess energy levels that enable efficient absorption of low-energy photons (typically in the NIR region) and subsequent emission of higher-energy photons (visible or UV). This often involves the use of lanthanide ions, such as erbium, ytterbium, and thulium, which exhibit multiple excited states suitable for UC.

#### Host Matrix

The material's host matrix should provide a suitable environment for the dopant ions to exhibit efficient UC luminescence. It should minimize non-radiative relaxation processes that can quench luminescence and maximize the probability of energy transfer between the dopant ions.

#### Dopant Concentration

The concentration of dopant ions within the host matrix needs to be optimized. Too low a concentration may result in inefficient energy transfer processes, while too high a concentration can lead to concentration quenching due to ion-ion interactions.

#### Pump Power Density

The efficiency of UC processes depends on the intensity of the excitation source. Higher pump power densities can lead to more efficient UC, but there's typically an optimal range where the UC efficiency is maximized without causing excessive heating or other detrimental effects.

#### Photon Absorption Cross-section

The absorption cross-section of the material at the excitation wavelength determines the efficiency of photon absorption. Materials with higher absorption cross-sections can efficiently absorb photons, leading to enhanced UC efficiency.

#### The Lifetime of Excited States

The excited states of the dopant ions should have sufficiently long lifetimes to allow for energy transfer processes and subsequent emission of higher-energy photons. Short-lived excited states may result in non-radiative relaxation pathways, reducing UC efficiency.

#### Stability and Compatibility

The material should be stable under the operating conditions and compatible with the intended application. This includes considerations such as chemical stability, thermal stability, and compatibility with fabrication techniques.

To unlock their full potential, synthesizing these materials with precise control over their size, morphology, and luminescence properties is crucial. Choosing the most suitable synthesis method depends on the desired properties of the UC material, the target application, and available resources. Each method has its strengths and weaknesses, and ongoing research continues to refine existing techniques and develop new approaches for optimizing the synthesis of UC materials. UC materials, typically luminescent nanoparticles, can be synthesized through various methods. Table 1 lists some of the most common methods for the synthesis of UC materials. Often, researchers combine different techniques or modify existing methods to achieve optimal results. Furthermore, ongoing research continues to explore novel synthesis approaches, such as hydrothermal-ionic liquid hybrid methods and biomimetic synthesis, aimed at overcoming existing limitations and further tailoring UC materials for specific applications.

**Table 1:** The most common methods for the synthesis of UC materials

Upconverter materials	Solvent	Reaction conditions	Post-heat treatment	Ref
Chemical precipitation				
MoO_3_: Yb^3+^/Er^3+^ NPs	Deionized water	90 °C, 0.5 h	750 °C, 2 h	[34]
NaYF_4_:Yb/Er@NaYF_4_:Nd/Yb NPs	Oleic acid, 1-octadecene in water	150 °C for 1 h	290 °C, for 1.5 h, Ar	[35]
Ln–Li NPs (Ln = Tm, Er, Ho) NaGdF_4_: Yb^3+^/Tm^3+^, NaGdF_4_: Li^+^/Yb^3+^/Tm^3+^ NPs	Octadecene (ODE), oleic acid (OA)	310 °C, 0.5 h, N_2_	-	[36]
Yb^3+^/Er^3+^ co-doped CaF_2_ NPs	Deionized water & alcohol	190 °C, 6 h	-	[37]
NaGdF_4_:Yb, Er NCs	Methanol	300 °C,1 h	-	[38]
Yb, Er co-doped YAG (YAG = Y,Al,Ga) polycrystalline	Ammonium hydrogen carbonate, alcohol, distilled water	80 °C for 48 h	1200 °C, 4 h	[39]
Mg_2_SiO_4_:Er^3+^ NPs	Ethanol, nitric acid	RT	800 °C, 0.5 h	[40]
La_2_Mo_2_O_9_:Yb,Er La_2_Mo_2_O_9_:Yb,Ho	Hydrochloric acid	900 °C, 8 h	-	[41]
ZnO:Er^3+^ NCs	Ethanol	80 °C, 1.5 h	300, 900 °C, 0.5 h	[42]
Solid state reaction				
Ba_2_LaNbO_6_ doped with Er^3+^ and Yb^3+^	-	1600 ◦C, 8 h, O_2_	-	[43]
Er^3+^ doped, and Er^3+^-Yb^3+^ co-doped La_4_Ti_9_O_24_ phosphors	-	800 ◦C, 6 h/1250 °C, 12 h	-	[44]
Cs_3_YF_6_: Er^3+^, Yb^3+^	-	600 C, 3 h	-	[45]
β-NaYF_4_: Er^3+^	-	650 ◦C, 4 h, N_2_	-	[46]
KLu_2_F_7_: Pr^3+^	-	800 °C, 3 h	-	[47]
LaNbO_4_:Er^3+^/Yb^3+^	-	1300 °C, 8 h	-	[48]
(YEr)_2_O_2_SO_4_ NPs	-	850 °C, 2 h	-	[49]
Yb^3+^–Er^3+^ co-doped Ba_3_Lu_2_Zn_5_O_11_ Yb^3+^–Ho^3+^ co-doped Ba_3_Lu_2_Zn_5_O_11_	-	1220 °C, 4 h	-	[50]
Tm^3+^/Yb^3+^ co-doped YTaO_4_ ceramic	-	1300, 1450 °C, 4 h	-	[51]
Er^3+^–Pr^3+^–Yb^3+^ tri-doped LaNbO_4_ phosphors	-	1100 °C, 4 h		[52]
Yb^3+^/Er^3+^ co-doped CeO_2_	-		1400 °C, 10 h	[53]
Yb^3+^/Ho^3+^ co-doped Lu_3_NbO_7_ phosphors	-	1400 °C, 6–12 h	-	[54]
Yb^3+^/ Er^3+^/ Ga^3+^ tri-doped ZnO	-	750 °C, 2 h	1200 °C for 8 h	[55]
GeO_2_-PbO-Na_2_O-Ga_2_O_3_-PbF_2_- Er_2_O_3_-Pr_2_O_3_ (GPNG glass)	-	1150 °C, 0.5 h	-	[56]
Sr_8_MgY(PO_4_)_7_:Yb^3+^–Er^3+^/Ho^3+^	-	1250 °C, 6 h	-	[57]
Ho^3+^/Tm^3+^/Yb^3+^ doped YTaO_4_	-	1300 °C, 5 h	-	[58]
Er^3+^ doped TeO^2+^ Na_2_O + ZnO + GeO_2_ + Er_2_O_3_	-	750 °C, 2 h	300 °C, 2.5, 5.0, 7.5, 10 h	[59]
Er^3+^ and Yb^3+^ co-doped KPb_2_Cl_5_		900 °C, 1 h	None	[60]
Melting quenching method				
Er^3+^ doped TeO_2_-PbCl_2_-WO_3_:Er^3+^	-	850 °C, 8 min	-	[61]
Er^3+^/Yb^3+^ co-doped (AlPO_4_ and Ca_3_(PO_4_)_2_)	-	1400 °C, 1 h	400 °C, 4 h/530 C, 5 h	[62]
NaYF_4_:Yb/Tm NCs	-	330 °C, 1 h	-	[13]
Er^3+^ doped Na_2_SO_4_–PbO–P_2_O_5_-Er_2_O_3_	-	900 °C, 10 min	300 °C, 2 h	[63]
Yb^3+^–Ho^3+^ co-doped TeO_2_–(BaF_2_ + BaO)–La_2_O_3_–Ho_2_O_3_–Yb_2_O_3_ Yb^3+^ doped TeO_2_–(BaF_2_ + BaO)–La_2_O_3_–Ho_2_O_3_–Yb_2_O_3_	-	700–750 °C	10 °C below the glass transition temperature, 2 h	[64]
Ag NPs/Er^3+^ co-doped Zinc Tellurite glass	-	800 °C, 3 h	-	[65]
Er^3+^ doped NaGdF_4_ NCs	-	1450 °C, 0.5 h	450 °C	[66]
Er^3+^ doped fluoroindate glasses	-	850 ºC, 20 min	240 ºC, 2 h	[67]
TeO_2_–Na_2_O–PbX glasses	-	900 °C, 20 min	500 °C	[68]
Solvothermal method				
NaYF_4_:Yb^3+^, Er^3+^	Deionized water, ethylene glycol	180 °C, 5 h	None	[69]
Tm^3+^, Gd^3+^, Sm^3+^ tri-doped KF–YbF_3_	Alcohol, oleic acid	210 °C, 20 h	None	[70]
NaYF_4_:Yb/Er/Gd NPs	Ethanol, Deionized water, oleic acid	150 °C, 7 h	-	[71]
NaYF_4_:Yb/Er/Ho/Tm	Ethylene glycol, H_2_O	190 °C,12 h	-	[72]
Er^3+^ /Yb^3+^ co-doped NaGdF_4_	1-octadecene, oleic acid	300 °C, 1 h, N_2_	-	[73]
Yb-Er-F tri-doped TiO_2_ NPs	Deionized water	200 °C, 24 h	500 °C, 2 h	[74]
Yb^3+^/Er^3+^ co-doped Ba(MoO_4_)(WO_4_) NCs	Deionized water	200 °C, 20 h		[75]
Er^3+^/Yb^3+^ co-doped TiO_2x_F_x_	Ethanol, H_2_O	200 °C for 24 h	500 °C, 2 h	[76]
SrF_2_:Yb^3+^, Ho^3+^, Er^3+^,Tm^3+^ NPs	Trisodium citrate, ammon.citrate in H_2_O	6–12 h	-	[77]
β-NaGdF_4_:Yb^3+^,Er^3+^ NRs	Ethanol, oleic acid, water	200 °C for 16 h	-	[78]
Ho^3+^-Yb^3+^-F tri-doped TiO_2_ NPs	Deionized water	200 °C for 24 h	450 °C, 0.5 h	[79]
Lu_2_TeO_6_:Yb^3+^/Er^3+^ nanophosphors	Deionized water	220 °C for 24 h	650–800 °C, 5 h	[80]
NaYF_4_:Yb,Er NPs	H_2_O, diethylene glycol (DEG)	180 ºC for 6 h	-	[81]
Thermal decomposition				
Er^3+^ and Yb^3+^ co-doped NaYF_4_	Conc. trifluoroacetic acid, oleic acid, oleylamine	275 °C for 30 min, Ar	-	[82]
Er^3+^ doped Y_2_O_3_ polycrystalline	-	1000 °C for 4 h	-	[83]
NaGdF_4_:Er^3+^/Yb^3+^ colloidal particles	1-octadecene, oleic acid	310 °C, N_2_	None	[78]
Combustion				
NaSrBO_3_: Eu^3+^/Tb^3+^ polycrystalline	Distilled water	550 °C for 4 h	600 °C, 700 °C, 800 °C	[84]
BaZrO_3_:Er^3+^ and BaZrO_3_:Er^3+^,Yb^3+^	Deionized water	550 °C for 5 min	1100 °C, 3 h	[85]
Sol–gel				
GdBO_3_:Yb^3+^/Tb^3+^	Water, nitric acid, ethyl alcohol, tetrabutyl titanate	120 °C	900 °C,3 h	[86]
Gd_2_(MoO_4_)_3_: Er phosphors	Deionized water, citric acid	130 °C, 20 h	800 °C, 2 h	[87]
Sr_3_Y_0.88_(PO_4_)_3_:Yb^3+^,Ln^3+^ phosphors (Ln = Ho, Er, Tm)	Aqueous solution, EDTA-NH_4_OH	300 °C	1250 °C, 4 h	[88]
Er_x_Yb_2x_Si_2_O_7_	-	400 °C, Ar/O_2_	1100 °C, 1 h, O_2_	[89]

It is clear that the hydrothermal and solvothermal techniques are the most suitable to control the reaction parameters (e.g. organic solvent, solvent volume ratio, dopant molar ratio, temperature, and reaction time) and hence tuning the particle size and morphology for targeted applications. In addition, the sol–gel method is useful for the production of high-quality UC luminescent nanoparticles with uniform and homogeneous distribution. It also offers high dispersion for liquids that can be used for efficient luminescent coating materials due to the high adhesion on the substrate. Moreover, the thermal decomposition route could be a proper process for the preparation of simple systems of UC materials with slight modifications in a short time.

### Structural Properties

**Fig. 6:**
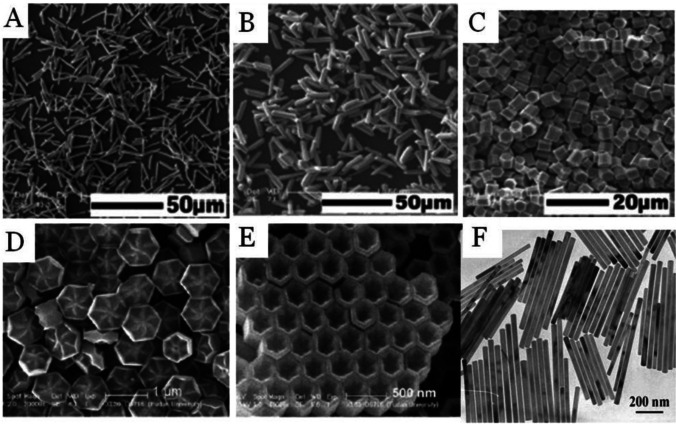
Different shapes of RE fluoride nano-/microcrystals by hydrothermal method [94]

It is well known that the excitation process and local temperature are the reasons for the luminescent colors of the optical materials [62, 90]. In addition, the incorporation of different doping ratios or multiple dopants during the chemical synthesis process could be a suitable way to tune the emission wavelength and overall emission intensity of the selected spectral peaks. For instance, NaYF_4_ is formed either in cubic or hexagonal structures. It is well known that a hexagon-shaped UC phosphor matrix has superior efficacy. However, most synthetic techniques result in the cubic phase of NaYF_4_ and NaYbF_4_ [91]. In addition, various morphologies of hexagonal NaYF_4_ structures, including prism, disk, tube, rod, and octahedral shapes were prepared via a hydrothermal route as shown in Fig. 6. A mechanistic investigation was conducted into the process of synthesizing various shapes of RE fluoride nano-/microcrystals. According to reports, the fluoride supply, pH level, and organic addition (e,g. trisodium citrate) have shown a significant impact on the forms [92, 93].

**Fig. 7:**
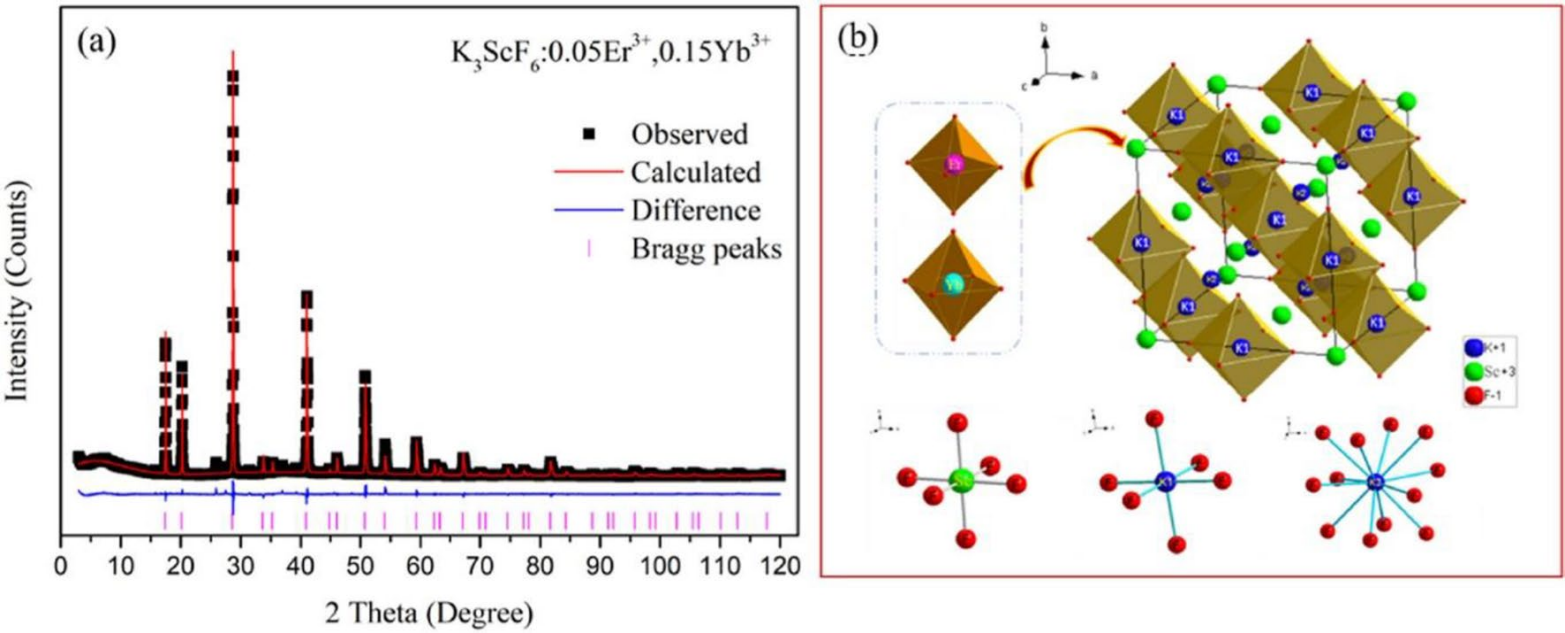
Crystal structure K_3_ScF_6_:Er^3+^, Yb.^3+^ as well as polyhedral coordination configuration of Er, Yb, Sc, K_1,_ and K_2_ with fluorine atoms [98]

To create well-shaped, monodispersed nanoparticles, thermal breakdown can be used to synthesize RE fluoride nanomaterials. Despite providing a tight size distribution and excellent shape control, the thermal decomposition approach is not without its shortcomings. High reaction temperatures (250–330 °C), organic solvents, surfactants, and an oxygen-free environment with inert gas protection are typically needed. [10, 95]. Accordingly, hexagonal NaYF_4_:Yb^3+^/Er^3+^ microcrystals obtained by thermal decomposition exhibited visible UC PL, which is 4.4 times higher than its cubic phase [94]. Compared to ZrO_2_ nanoparticles in the tetragonal phase, monoclinic ZrO_2_ nanoparticles emit a greater UC PL. To achieve an effective UC process, it is crucial to choose a suitable host lattice with a low phonon cutoff energy and low crystal field symmetry [10, 96]. The Fluoride compound with the general formula of A_3_BF_6_ (A = Li, Na, K, NH_4_, etc.; B = Al, Sc, V, Cr, Fe, Y, Ga, etc.) is crystallized in the cryolite structure, and considered as one of the most promising UC host in the field of luminescent materials owing to its low phonon energy, stable chemical composition, and good optical transparency. Recently, compounds with cryolite structures have been widely used as hosts for luminescent materials, such as K_3_GaF_6_:Mn^4+^, Na_3_GaF_6_:Mn^4+^, K_3_LuF_6_:Er^3+^, K_3_LuF_6_:Ce^3+^, Na_3_AlF_6_:Mn^4+^ red phosphor was prepared via the coprecipitation and hydrothermal methods. The prepared phosphor was known as a promising candidate for application in warm WLEDs. Clearly, the emission light from K_3_LuF_6_:Tb^3+^, Eu^3+^ phosphors can be tuned from green to yellowish pink by adjusting the concentration ratio of Eu^3+^/Tb^3+^ [97]. High-temperature solid-state synthesis was used to prepare the Yb^3+^/Er^3+^ co-doped K_3_ScF_6_ phosphors, and X-ray diffraction (XRD) was used to confirm the crystal phase. XRD represents a model of the K_3_ScF_6_:Er^3+^, Yb^3+^ as well as polyhedral coordination configuration of Er, Yb, Sc, K1, and K_2_ with fluorine atoms, as displayed in Fig. 7 [98].

**Fig. 8:**
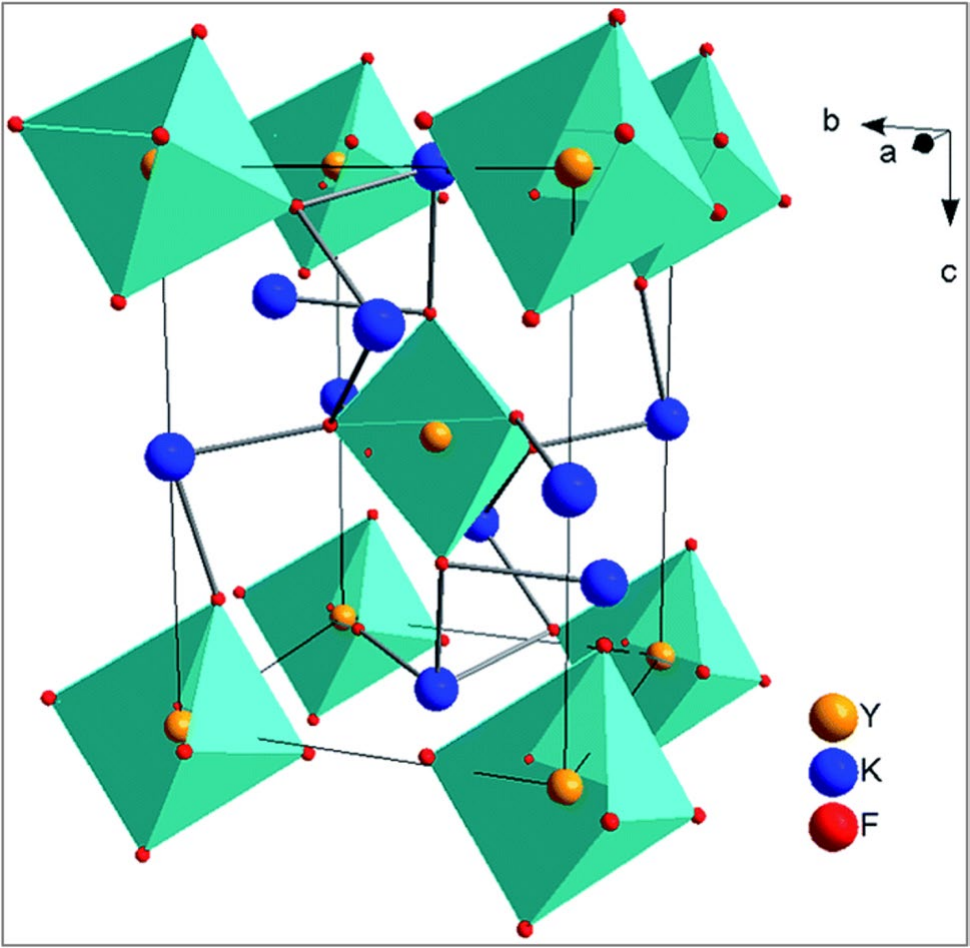
Crystal structure of monoclinic K_3_YF_6_ [99]

The cryolite salt of K_3_YF_6_:Er^3+^, Yb^3+^ was prepared by a high-temperature solid-state method. The X-ray diffraction (XRD) analysis confirmed the polyhedral coordination configuration of Er, Yb, Sc, K_1_, and K_2_ with fluorine atoms [98]. It was discovered that the crystal structure of K_3_YF_6_ is monoclinic, with Y^3+^ firmly occupying the deformed octahedral sites that have six-fold coordinated fluorine ions and centrosymmetric Ci local symmetry, as depicted in Fig. 8. It is thought that during the doping process, Yb^3+^/Er^3+^ replaces the site for Y^3+^ because of their near proximity in ionic radii and electricity valences [99].

Highly pure green light emission from Er^3+^ and Yb^3+^ doped La_4_Ti_9_O_24_ has been reported; this could find applications in advanced domains such as biomedical, sensing, and 3D printing. [62]. Er^+3^ and Yb^+3^ doped La_4_Ti_9_O_24_ were reported to provide an extremely high purity of green light emission that could be utilized in various advanced fields like sensing, biomedical, and 3d printing [62].

## Strategies for Enhancing Upconversion Luminescence

Luminosity thermal quenching often affects luminescent materials as a result of different non-radiation relaxations becoming worse at higher temperatures [100]. Various materials have been recognized as upconverters in several superior fields, however, the current upconverting materials, are still too ineffective for sustainable implementation. In this viewpoint, significant improvements could enhance the upconversion proficiency. These developments give the upconversion materials the ability for further applications and efficiency [101]. Extensive research work has been performed for upgrading the upconverting materials, however, it is still facing many limitations including spectral alignment, bandwidth, size phase, and low efficiency. Successive enhancements are recommended to enhance the upconverter's efficiency and address properties comprising the host material, elemental doping, and surface features. For example, upconverters utilize photonic crystals, antenna dyes, quantum dots, and plasmonic parts showing superior performance [102]. Additionally, there are significant lack of information about the quantum yield of the provided upconverters in the reported studies. Therefore, it is hard to identify the competence of UC material in optoelectronic and solar cell devices, and it is difficult to compare them due to a lack of data on its quantum yield [103].

**Fig. 9:**
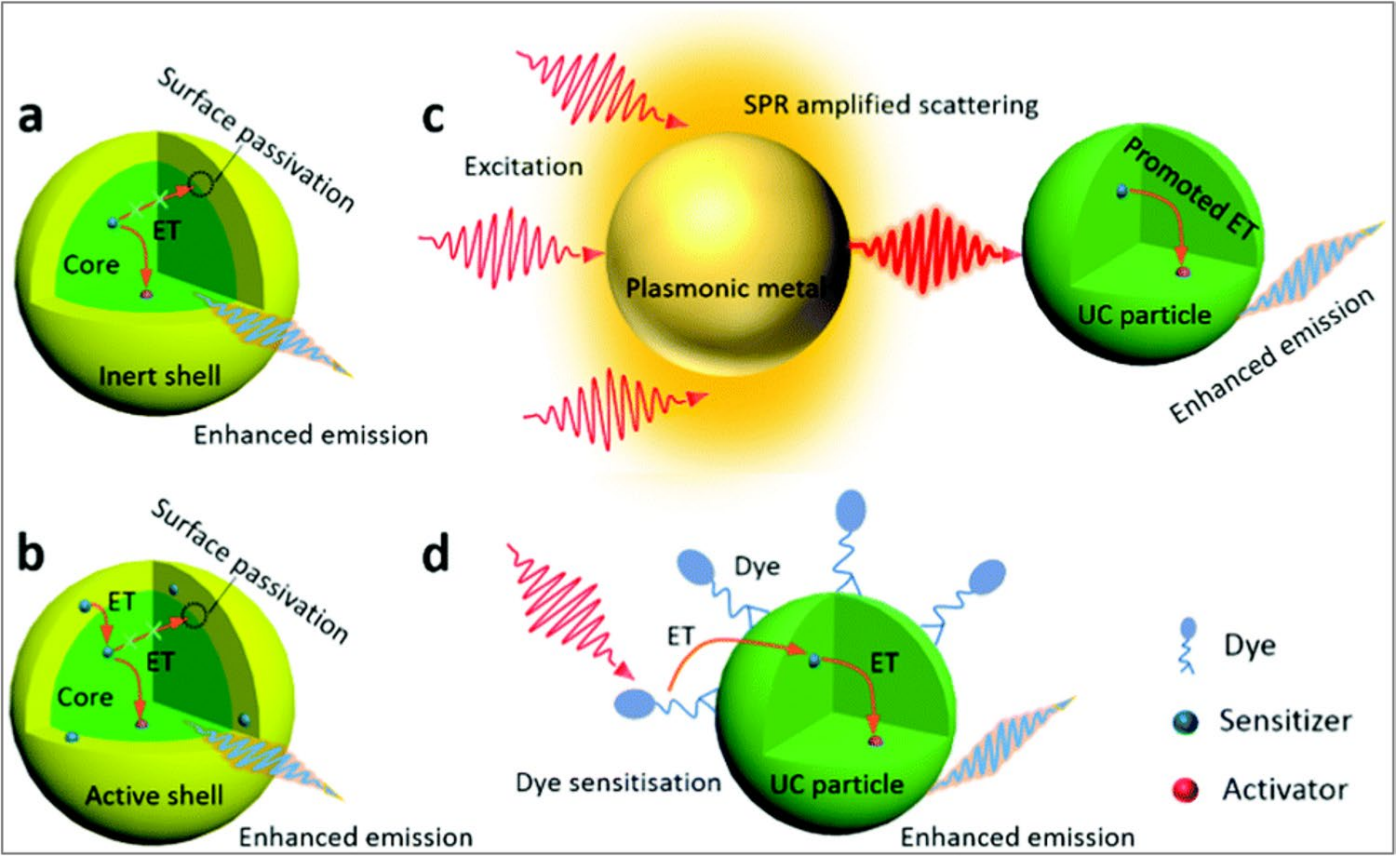
Various strategies to enhance UC emission efficiency [9]

Various strategies have been studied for the enhancement of the upconverter materials and the whole UC process. In this review four main strategies, (a) active core/inert shell structure, (b) core/active shell structures, (c) coupling with plasmonic structures, and (d) sensitization using synthetic dyes, as discussed below with examples. Figure 9 demonstrates a schematic diagram for these four strategies [9].

### Constructing Active-core@inert-shell Structures

**Fig. 10:**
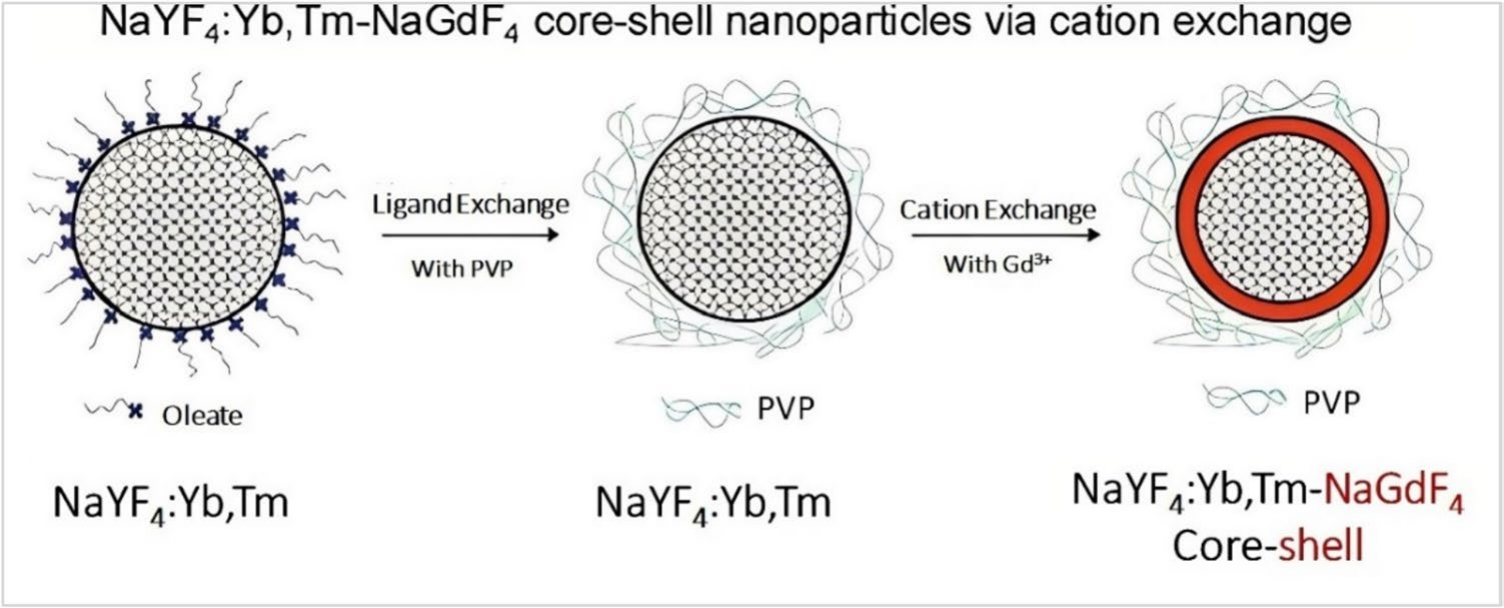
Schematic design for preparation of core@inert-shell structure [107]

On way to enhance the quantum yield of upconverting materials is to construct core@inert-shell. This assembly does not contain any dopants, which reveals an inert behavior of the inert shell [9, 104]. Recently, NaYF_4_:Yb/Er@NaYF_4_ NPs with 45 nm size were prepared with an inert shell, which revealed a quantum yield value equal to 9% in the NIR spectrum [9]. Another example, NaErF_4_:Tm@NaYF_4_ nanoparticles were synthesized by coating the NaYF_4_ shell on a core of Er-enriched NaErF_4_:Tm. This new structure improved the emission from 654 nm to reach the value of 708 nm by suppressing the fixed quenching compared with that of the UC core [105]. NaErF_4_@NaLuF_4_ core@shell nanocrystals were synthesized, measured, and deliberated to be the greatest NIR-to-visible with a quantum yield of 5.2% which was greater than that of the core only by 57 times at 10 W/cm^2^ of irradiation source. [106]. In addition, β-NaYF_4_:Yb, Er@β-NaYF_4_ core/shell structure was synthesized by the hot-injection method by controlling the core size and shell thickness via adjusting the amount of the stabilizer and precursors addition [106]. The upconversion performance was reported to be improved by 100 times in comparison with the original nanocrystals. Another Active core with a uniform inert shell was investigated for the Yb^3+^, Tm^3+^ codoped NaYF_4_ host nanoparticles with a uniform NaGdF_4_ shell as schematically represented in Fig. 10. Through this configuration, the upconversion enhancement could be easily achieved by combining the paramagnetic property of Gd^3+^ and the sensitizing effect of Yb^3+^ and Tm^3+^ ions to be applied in biomedical imaging [107].

### Constructing Active-core@active-shell Structures

Another approach to enhance the UC photoluminescence efficiency is to support it with an active shell [108]. The use of this approach has been extended to numerous UC particles. Yb^3+^ doped active shell was found to improve NIR energy transfer efficiency and UC photoluminescence intensity. NaYF_4_:Yb/Er@NaLuF_4_:Yb/Tm represents an example of an active core–shell structure that achieved a significant impact on the UC photoluminescence performance [288. The epitaxial growth of the NaLuF_4_:Yb/Tm shell significantly inhibited the surface quenching influence leading to a 30% improvement in the photoluminescence intensity of a single core/shell particle matched with that of NaYF_4_:Yb/Er [109]. NaYF_4_:Yb, Er@NaYF_4_:Yb@ NaNdF_4_:Yb@NaYF_4_:Yb core-multi-shell UC nanoparticles displayed an entire quantum yield of 0.18% at 540 nm [9].

**Fig. 11:**
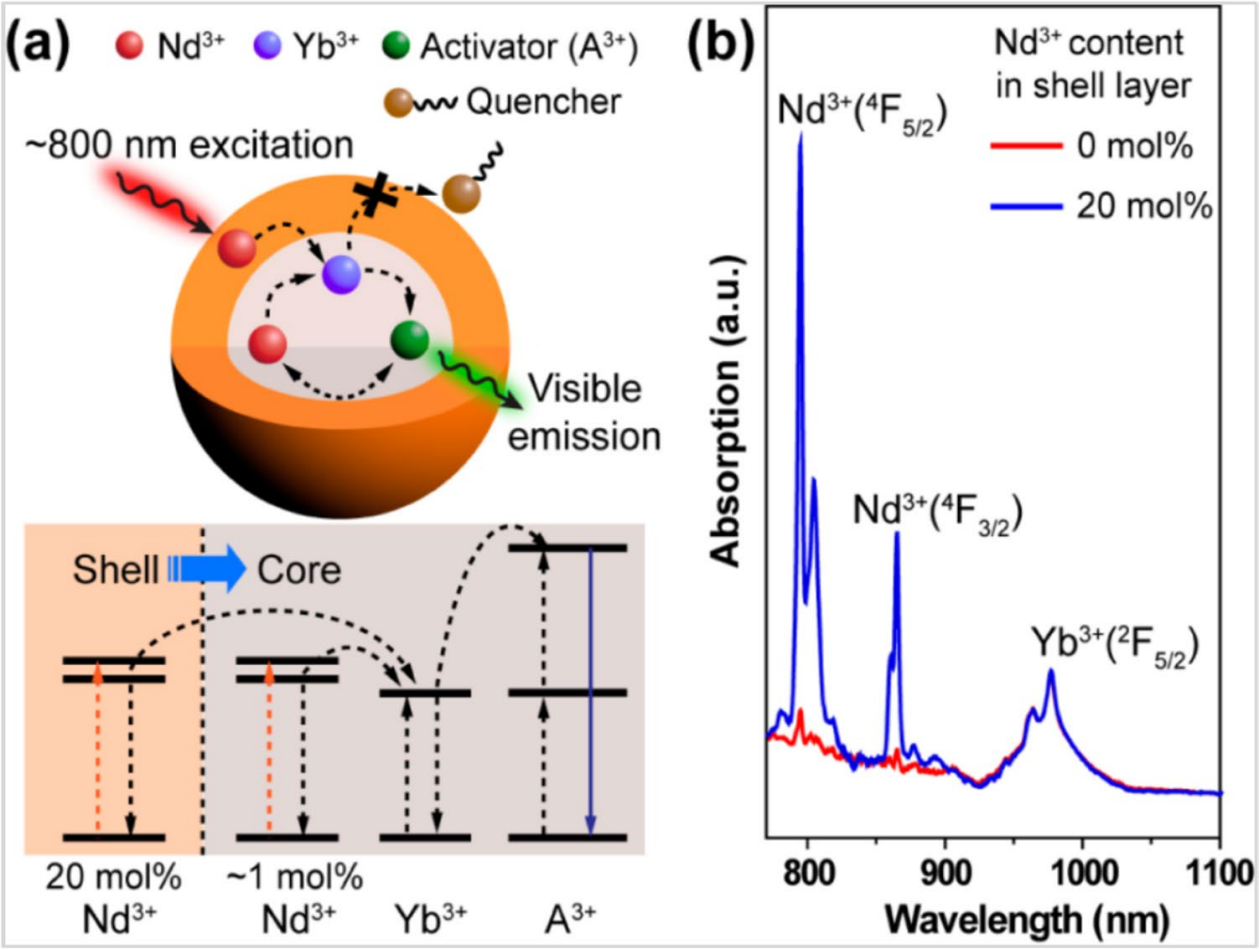
**a** Schematic design (top) and simplified energy level diagram (bottom) of a core–shell nanoparticles for photon upconversion under 800 nm excitation. **b** Near-IR absorption spectra of NaYF_4_:Yb/Nd (30/1%) nanoparticles coated with an inert NaYF_4_ shell or an active NaYF_4_:Nd(20%) shell [111]

The obtained quantum yield was 13 times greater than that of NaYF_4_:Yb/ Er@NaYF_4_:Yb@NaNdF_4_:Yb nanoparticles. The combination of up and down conversion luminescence in one system was also achieved by combining the NaYF_4_:Er^3+^, Yb^3+^ core with the CsMnCl_3_ shell perovskite heterostructure [110]. The obtained assembly possessed a change in the emission color with varying the excitation source according to the depth of penetration of the incident radiation, producing various luminescent configurations. This also helped in production of the anti-counterfeiting materials with outstanding performance [100]. Er^3+^, Yb^3+^ doped NaGdF_4_ nanoparticles prepared with improved thermal decomposition display luminescence emissions in the visible spectrum when excited with NIR light energy [107]. The quenching effect of the luminescence was minimized by coating the nanoparticles with a shell of NaGaF_4_. These coated particles showed a superior intensive luminescence emission in comparison with the uncoated particles revealing the impact of the active core shell structures. The balanced core–shell strategy was also studied in Yb^3+^/Nd^3+^ codoped NaYF_4_ nanoparticles and explained in the schematic design in Fig. 11. Typically, Nd^3+^ ions served as a sensitizer to transfer the energy to the Yb^3+^ ions. In this matrix, Nd^3+^ was doped in lower concentration to the core particles to minimize the concentration quenching and with a high concentration to the shell for proficient light harvesting. The overall result is enhancing the upconversion emission and providing a highly attractive luminescent performance for bio-imaging compared with the singly Yb^3+^ doped nanoparticles [111].

## Coupling with Plasmonic Metals

**Fig. 12:**
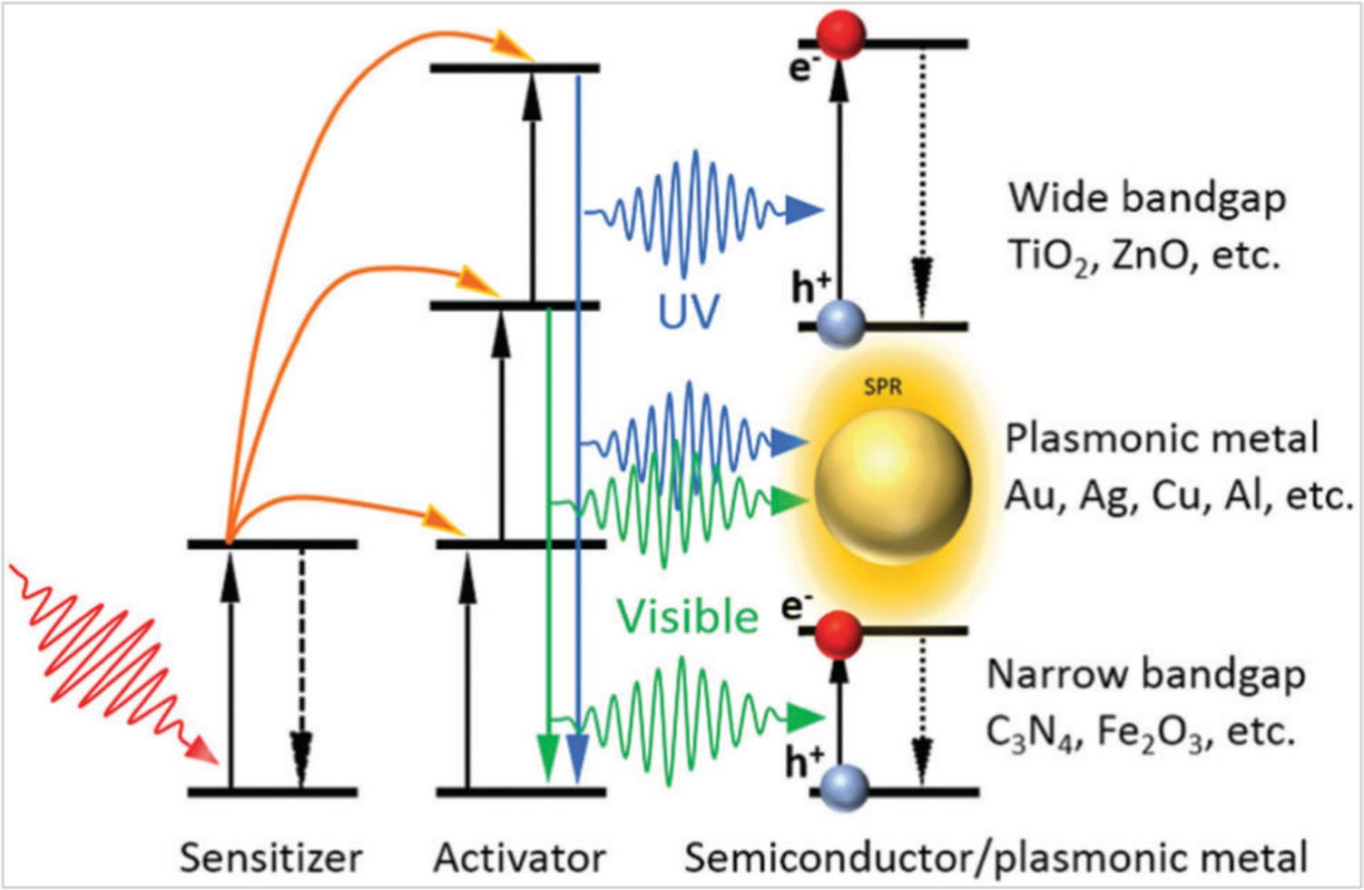
Schematic diagram for UC enhanced photocatalysis via plasmonic metals [9]

Many research studies reported that the photoluminescence performance could be improved by coupling the upconverters with the surface plasmon resonating body [112, 113]. Various strategies have been developed to adjust and control the interaction between the upconversion materials and the plasmon metal. The combination between the upconverter and plasmon can be performed through direct close contact in core@shell or layered configurations, as shown in Fig. 12 [9].

**Fig. 13:**
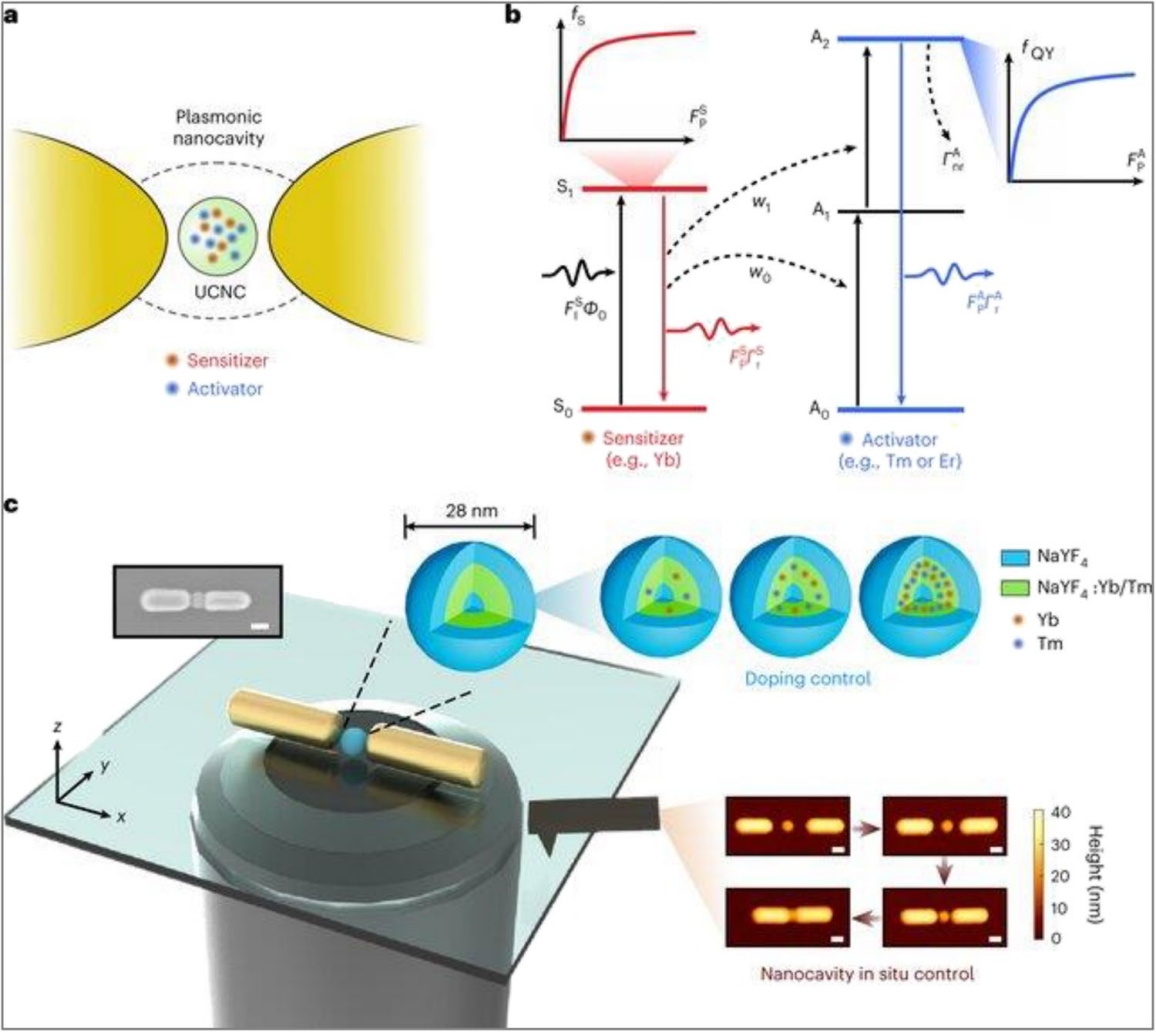
**a** The integration and coupling of plasmonic metal with upconverter materials using a third party [111], (**b**) Real image for the gold plasmon-Enhanced Upconversion [102, 115]

In such a strategy, photoluminescence is enhanced in two different ways, the first is internal by improving the inner structure of the upconversion material like composition, additives, crystal, and surface features and the second is external by plasmonic coupling. Plasmonic metal and upconverter units’ configuration could also be integrated with a third material like TiO_2_, SiO_2_, and graphene forming a link between the two components [114]. The internal and external modifications were studied and reported for NaYF_4_ doped with Yb and Tm to enhance their upconversion efficiency to the required level of applications [114]. This study revealed 2.3 × 10^5^ times enhancement in the photoluminescence as displayed in Fig. 13a. Silver nanowires were deposited on a monolayer of upconverting material, which led to 10 times enhancement of the UC results compared with the standard sample [9]. On the other hand, gold nanorods were used as plasmon for selectively enhancing the emissions of Er-doped NaYF_4_. Plasmonic improvement was utilized to enhance the NaYF_4_ nanophosphors doped with Yb^3+^ and Er^3+^ by coupling with dimensionally tailored Au nanorods [102]. This configuration attained superior luminescence emissions of several-fold enhancement, as displayed in Fig. 13b [102, 115]. Such a new approach for modulating UC emission via plasmonic coupling of the Au nanoparticles recorded multiple times improvement in the UC luminescence intensity because of plasmonic interaction [115].

### Dye Sensitization Process

**Fig. 14:**
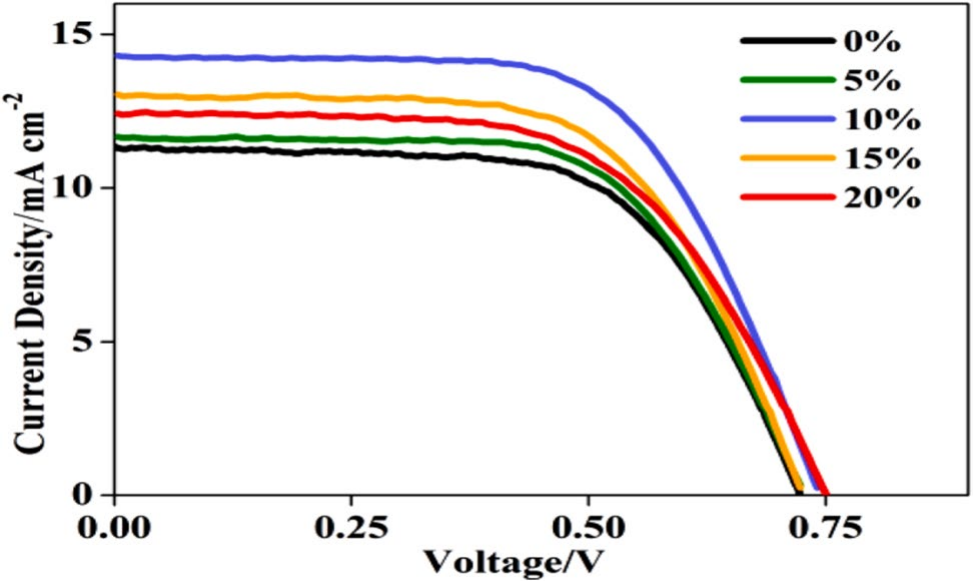
I–V curves of DSSCs containing P25 photoanode films with different Er@Nd@SiO_2_ dopant concentrations [118]

Such a strategy emerged from the fact that the NIR dyes possess a greater extinction coefficient and wider absorption range in comparison with the Ln^3+^ ion sensitizers. Examples of these synthetic dyes are IR 806, IR 808, IR 820, IR 845, indocyanine green, and Cyto 840 [116, 117]. It is found that IR-808 dye can be used to obtain a superior quantum yield like that of NaYbF_4_:Tm@NaYF_4_:Nd core/shell structure [9]. Researchers studied the coupling of upconverting material and TiO_2_-HSs to be applied to enhance the PV performance of DSSCs but still uncommon [118]. In this study, nanoparticles of NaYF_4_:Yb, Er@NaGdF_4_:Nd@SiO_2,_ and TiO_2_-HSs were utilized as upconverting material and scattering layers, respectively. Results illustrated that the dye-sensitized solar cells (DSSCs) could achieve about 29.63% improvement in the PV efficiency compared with the cell without the upconverting material as shown in Fig. 14 and Table 2.

**Table 2:** Photoelectric parameters of the DSSCs containing P25 photoanode films incorporating the TiO_2_-HSs and TiO_2_-HSs:10% Er@Nd@SiO_2_ UCNPs photosensitized by dye N719 [118]

Sample	J_SC_ [mA cm^−2^]	V_OC_ [V]	FF	PCE [%]	Dye laoding [mol cm^−2^]
TiO_2_-HSs	14.80	0.74	0.63	6.97	1.29 × 10^–7^
TiO_2_-HSs:10% UCNPs	17.78	0.75	0.61	8.17	1.91 × 10^–7^

## Applications of Upconversion Materials

**Fig. 15:**
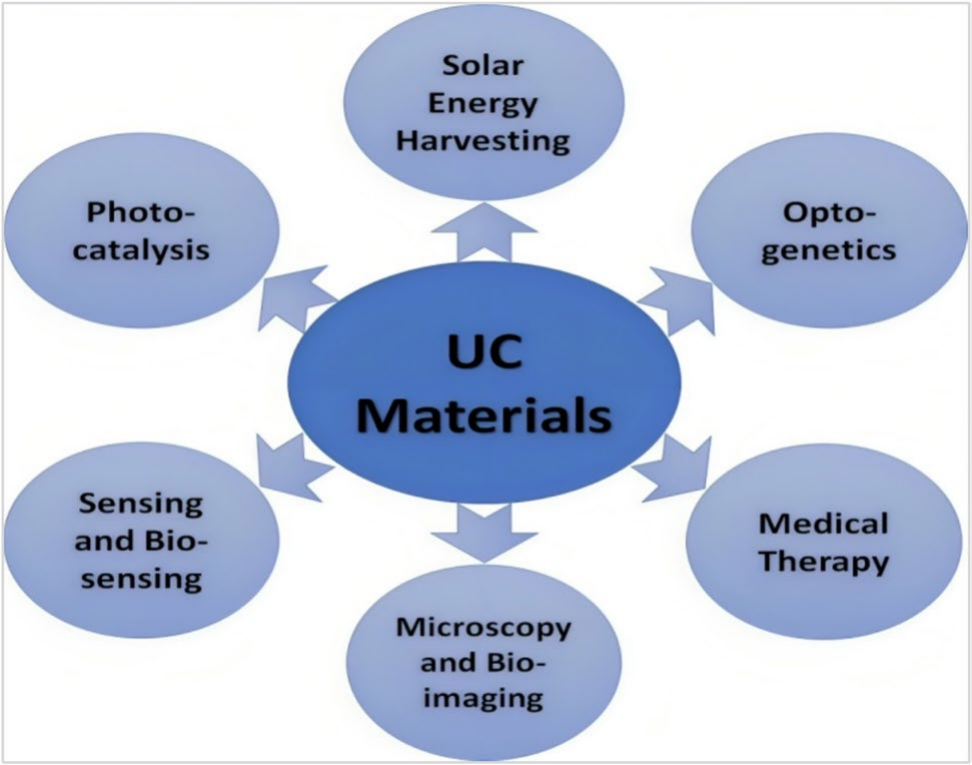
Various applications for UC materials

The UC materials have been employed in various advanced applications such as solar cells and photocatalysis [5, 9]. Meanwhile, lanthanide-doped UC materials have attracted wide concerns in medical and biological fields, such as biosensing, optogenetics, display technologies, bioimaging, photodynamic, and photo-thermal therapies, as shown in Fig. 15.

### Solar energy Harvesting

To achieve the maximum performance of harvesting solar light, UC units should be able to (i) absorb light in a broad light spectrum, (ii) have a high quantum yield, especially under low intensity and incoherent illumination, and (iii) possess high photostability since it will act with the solar cell components under solar radiation with temperature variations. The effect of upconverters with a small bandwidth is beneficial to increase the Shockley–Queisser efficiency limit for solar cells with a band gap equal to 1.7 eV from 28% to about 44 by using the sub-band gap photons in the IR bands converting it into photons with higher energy in the visible region [5]. It’s known that PV solar cells do not absorb the photons with energy lower than the band gap of the active material, which leads to wasting a considerable amount of solar energy [119]. For example, the organic chromospheres satisfied these requirements and were applied in light-gathering for PV and photocatalytic purposes [118]. The upconverter works to harvest this non-utilized sub-threshold energy beyond the solar cells. This produced photons with higher energy redirected to the solar cell in suitable bandwidth and absorbed by the solar cell harvesters [120]. A combination of UC nanocomposite (NaYF_4_: Yb, Er@NaGdF_4_:Nd@SiO_2_) with TiO_2_ was investigated to improve the performance of dye-sensitized solar cells (DSSCs). The photoelectric efficiency was enhanced by 29.63% through a dual mechanism between the upconverter and the TiO_2_ host material [121].

**Fig. 16:**
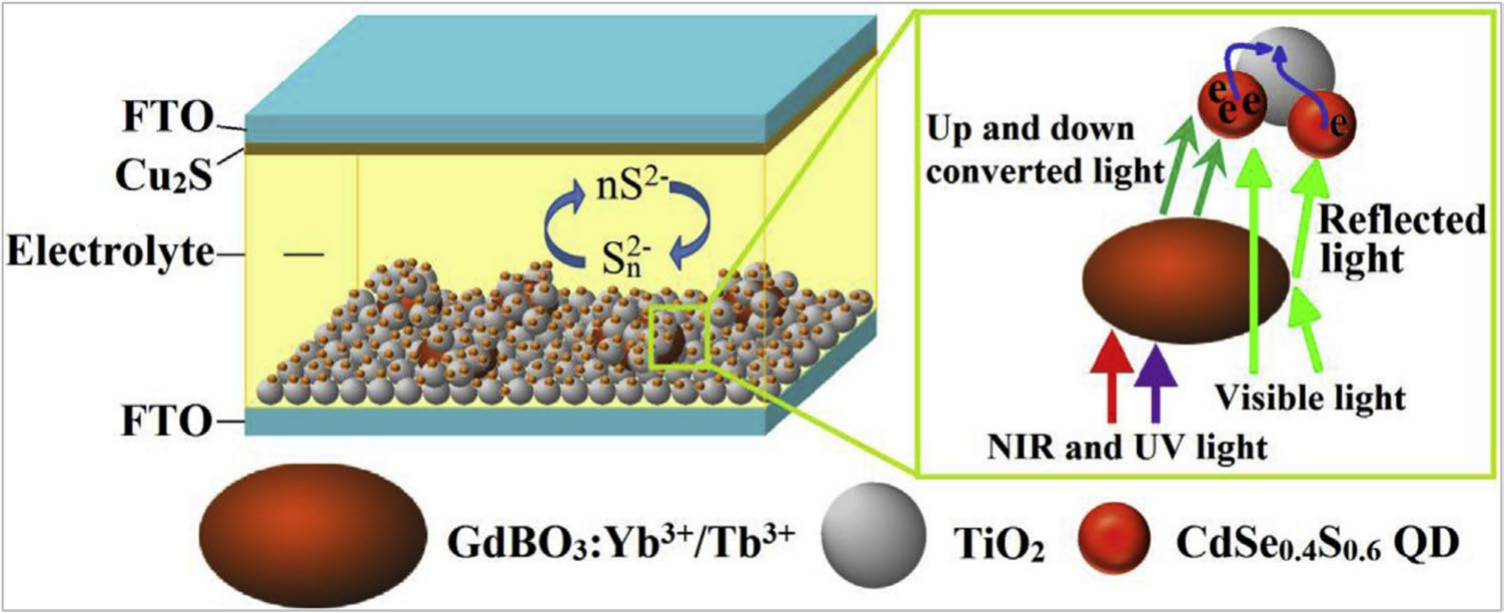
Schematic illustration of CdSe_0.4_S_0.6_/(GdBO_3_:Yb^3+^/Tb.^3+^@TiO_2_) photoanode in CdSe_0.4_S_0.6_ QDSSCs [86]

New upconversion materials (GdBO_3_:Yb^3+^/Tb^3+^) were also utilized to improve the light capture ability of the CdSeS quantum dot-synthesized solar cells (QDSSCs). The results demonstrated that the light absorption capability of the active material (CdSeS) was enhanced after the addition of GdBO_3_:Yb^3+^/Tb^3+^ phosphor in the solar cell displaying the highest PV conversion efficiency as shown in Fig. 16 [86].

**Fig. 17:**
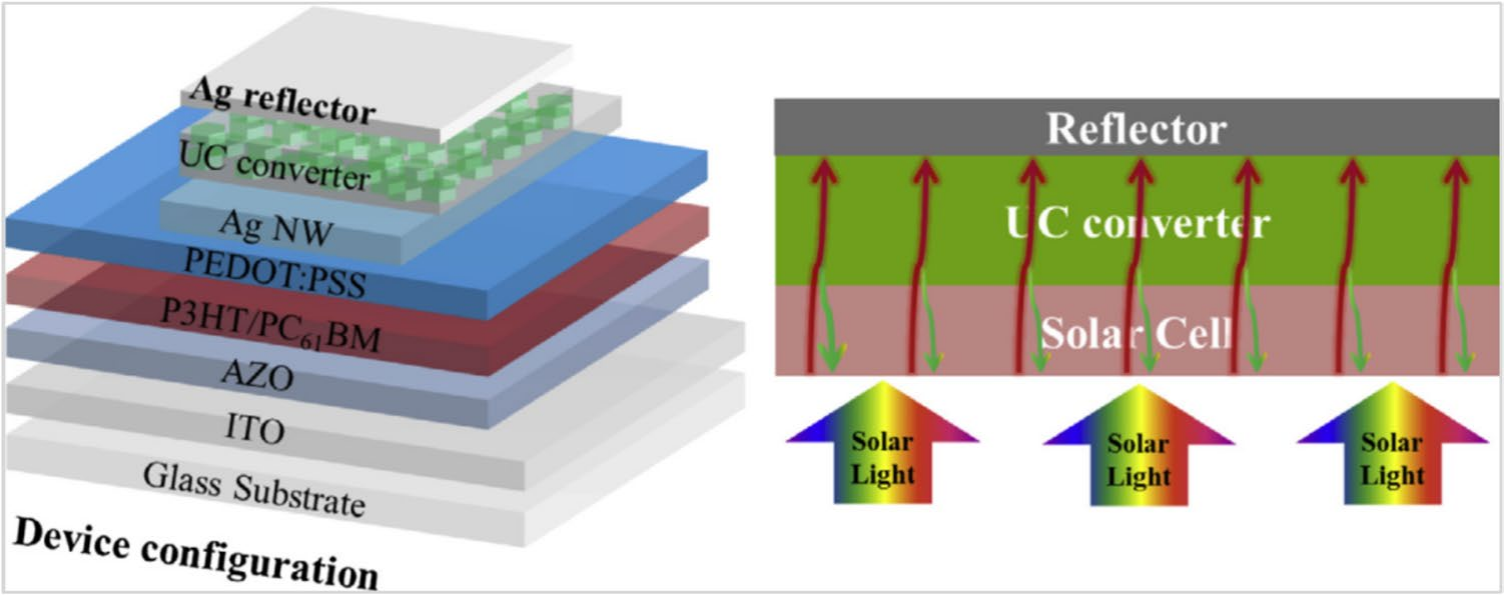
Configuration of semitransparent organic solar cells with lanthanide-doped UC converter (NaYF_4_: Yb/Er (20/2 mol%) [122]

Another group of scientists studied the integration of Yb/Er-doped NaYF_4_ layer in the organic solar cells (OSCs). The optical characteristics and measured photocurrent of the integrated solar system were clearly enhanced even under NIR illumination. The reason for such improvement is simply clarified by the role of Yb/Er-doped NaYF_4_ phosphor which converts the photons of the sub-band gap energy to the visible light range where it can be effectively absorbed by the solar cell, as shown in Fig. 17 [122]. Obviously, the solar cell receives light from the sun from the ITO electrode side. Sub-bandgap photons can go through the solar cells and into the UC converter, where they can undergo an up-conversion process to become visible photons. The solar cell can then absorb the transformed visible photons and create photocurrents.

**Fig. 18:**
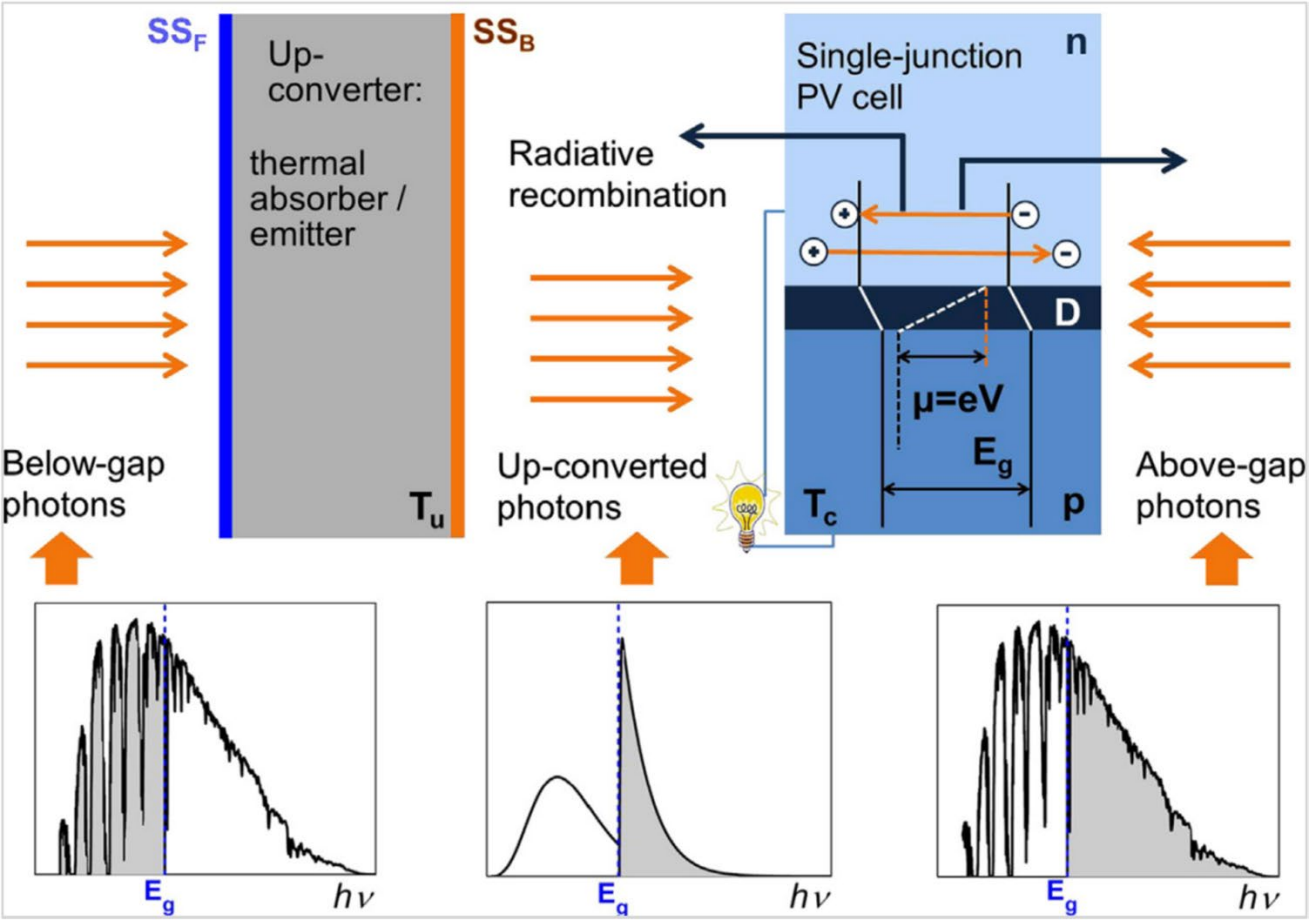
Schematic of the thermal up-conversion concept [123]

Solar light was also harvested based on the thermal upconversion process by developing a selective upconverter to exploit the thermal waste of the low-energy photons. The solar system consisted of the back and front sides of a single-junction PV cell and a solar-thermal up-converter compatible which is called a hybrid TPV. This type of arrangement offered light harvesting over the S-Q limit reaching 73% conversion efficiency of non-concentrated sunlight as schemed in Fig. 18 [123]. Photons of sunlight with energy above the bandgap enter the PV cell through one surface, while photons generated by the upconverter with energies above the bandgap enter through the other surface. The upconverter is a solar absorber/emitter with spectrally and angularly selective front surface emittance (SSFE) and back emittance (SSBE). The sculpted thermal spectrum of radiation radiated from the up-converter back surface and the energy spectra of terrestrial solar radiation (AM1.5D) are displayed in the insets.

### Photocatalysis

**Fig. 19:**
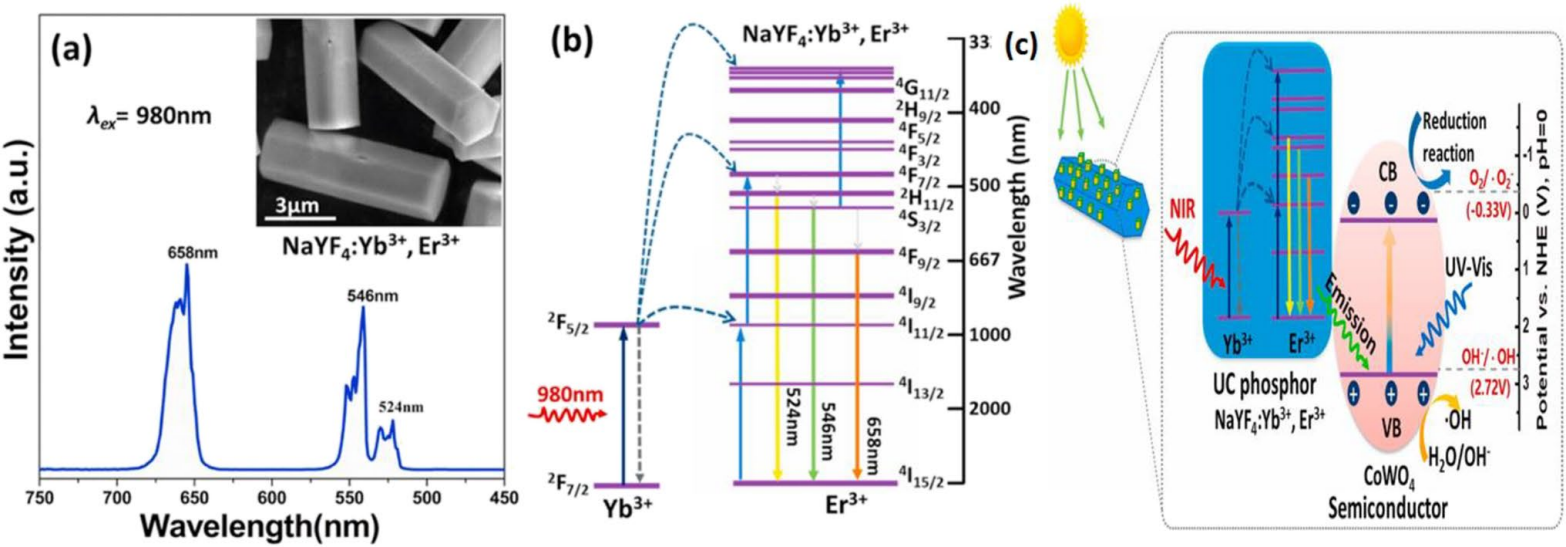
Illustration of the UC enhanced photocatalysis [126]

Another important application of the UC materials is photocatalysis which can be implemented in different fields like water splitting [123], water treatment [124], nitrogen photo-modification [125], and photo-electrocatalytic applications [126]. New composite material CoWO_4_@NaYF_4_:Yb^3+^, Er^3+^ was developed and evaluated as a catalyst for the photodegradation of organic dyes under visible and NIR irradiation. These novel composites greatly improved the photocatalytic activity by 87% compared with the pristine CoWO_4_ based on the upconversion mechanism as shown in Fig. 19 [127]. One way to achieve these targets is by increasing the photosensitivity of the photocatalytic substrate in the NIR spectrum region. Various semiconductors and lanthanides doped composites have been reported for enhancing the photocatalytic performance through diverse mechanisms mainly the TTA, and spectrum upconversion [127]. On the other hand, NaYF_4_:Yb^3+^, Tm^3+^ upconverter was applied on the surface of Ag_3_PO_4_@BP (black phosphorus) composite for photocatalytic applications in H_2_ production. The composite with the upconverter part exhibited noticeably improved photocatalytic performance in H_2_ evolution at a ten times rate than the BP alone [127]. The UC application of NaYF_4_ doped with Yb^3+^ and Tm.^3+^ gives Ag_3_PO_4_@BP the ability to utilize the light with lower energy especially at 980 nm instead of being wasted [128]

### Sensing and biosensing

**Fig. 20:**
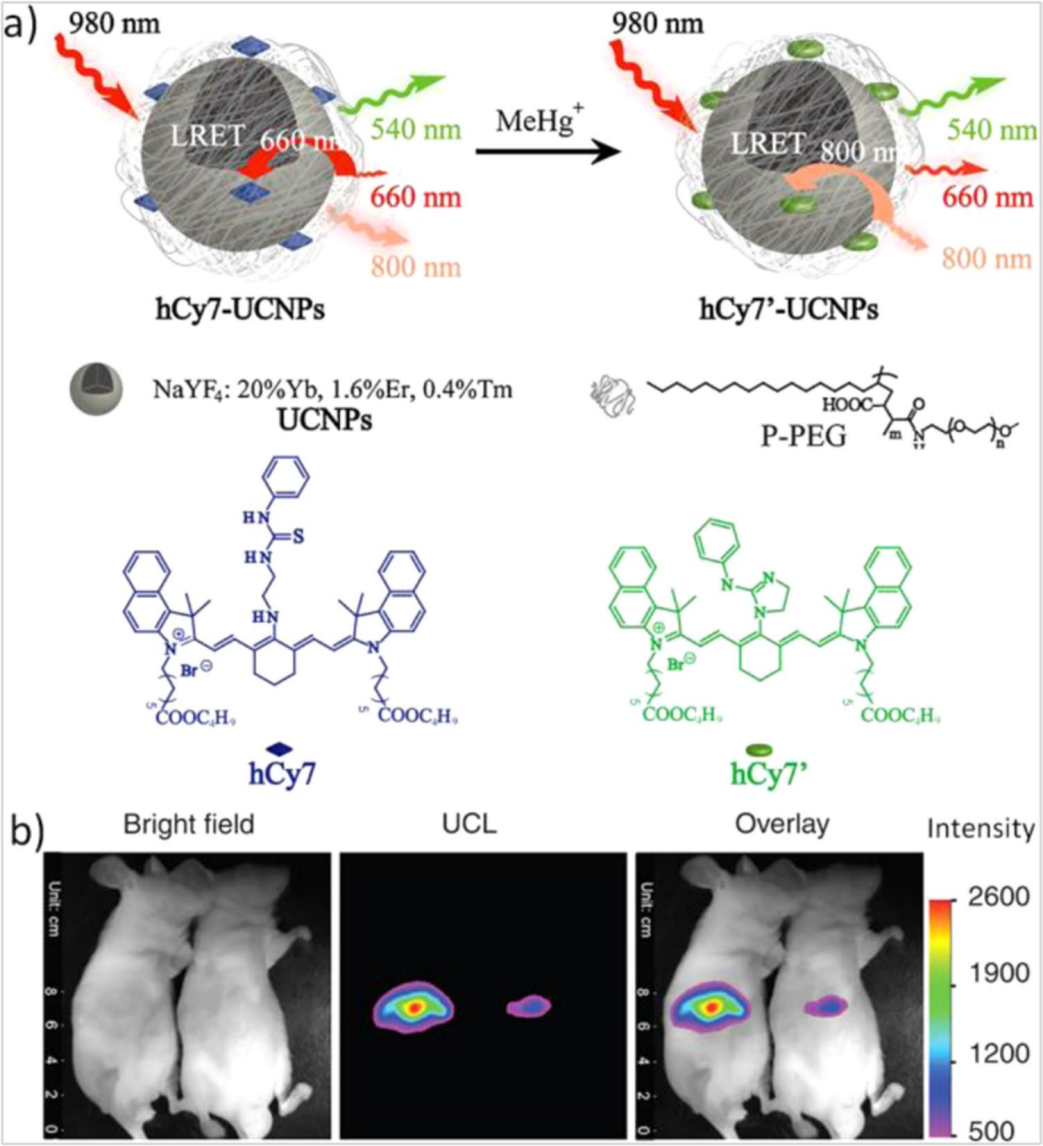
**a** Schematic illustration of upconversion nanosystem for MeHg^+^ detection. **b** In vivo detection of MeHg.^+^ in a mouse [7]

The recent progress in UCNPs synthesis has enhanced their photoelectric and photocatalytic applications along with biomedical applications like biosensing, bioimaging, optogenetics, drug release, and photodynamic thereby especially for UCNPs with low irradiance NIR excitation at wavelengths matching the biological window [5]. Upconversion luminescence (UCL) Ba_2_LaNbO_6_ phosphor doped with Er^3+^ and Yb^3+^ was investigated as a temperature sensor because it has a double perovskite structure, that can demonstrate two emission capabilities in the green and red spectrum [43]. Er^3+^ and Yb^3+^ doped Cs_3_YF_6_ were also studied for temperature sensing and green light display because release obvious green light under suitable excitation wavelength [45]. Lanthanide-doped upconversion nanophosphors possess large anti-Stoke shifts, low excitation energy, long lifetime of the emitted luminescence, strong tissue penetration, thin emission bandwidths, and absence of auto-fluorescence in the NIR light excitation. This property gives these materials the ability to transfer the energy from the excited donor to an acceptor component reducing the fluorescence emission intensity in the donor to the acceptor, in phenomena called resonance energy transfer, which in concern allow the using of these materials in the biosensing fields like the detection of many analytes biologically Fig. 20 [7].

**Fig. 21:**
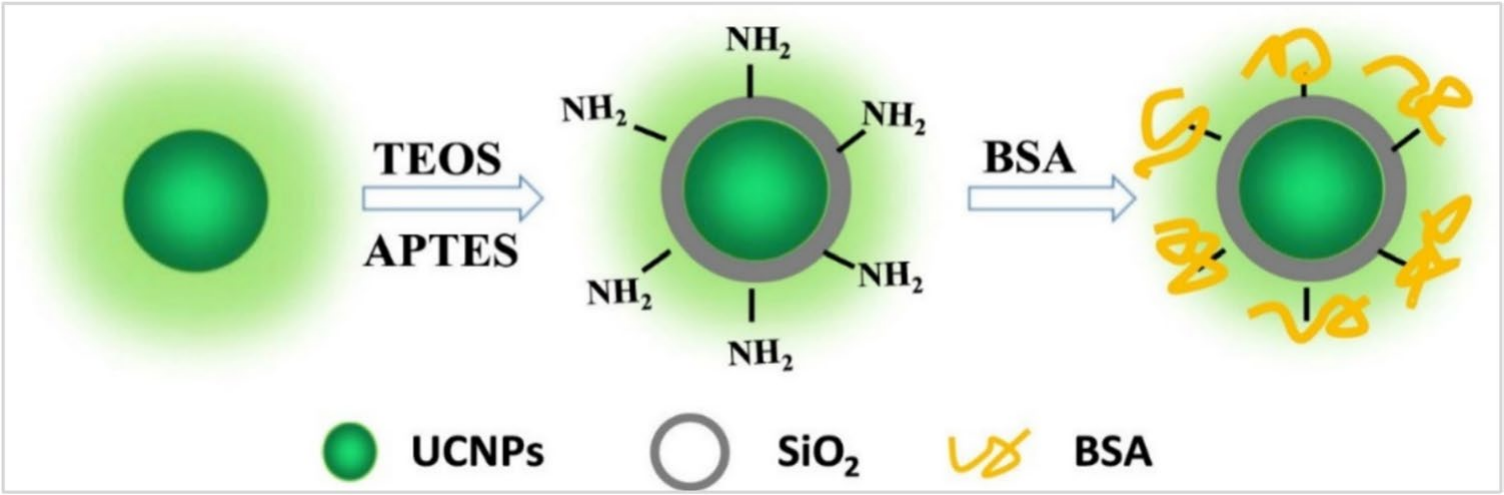
Schematic illustrations for the preparation of the UCNPs@SiO_2_@BSA composites [105]

Silica and BSA-coated upconversion nanoparticles (UCNPs@SiO_2_@BSA) in the core shell-shell decoration, were reported as biosensor composites for pH changes through pH-sensitive days and ranges as displayed in the scheme in Fig. 21 [127]. This type of pH sensor displayed a higher sensitivity compared with other similar materials.

Thiazole derivatives supported with UCNPs were established and tested as radiometric signals for mercuric ions in cellular biosystems at definite wavelengths in which the upconverters show better photo stability and high selectively. This study confirmed that the upconversion materials in the nanosized scale should be a futuristic strategy for the detection of toxic and harmful metal ions in living cells [129].

### Microscopy and Bioimaging

**Fig. 22:**
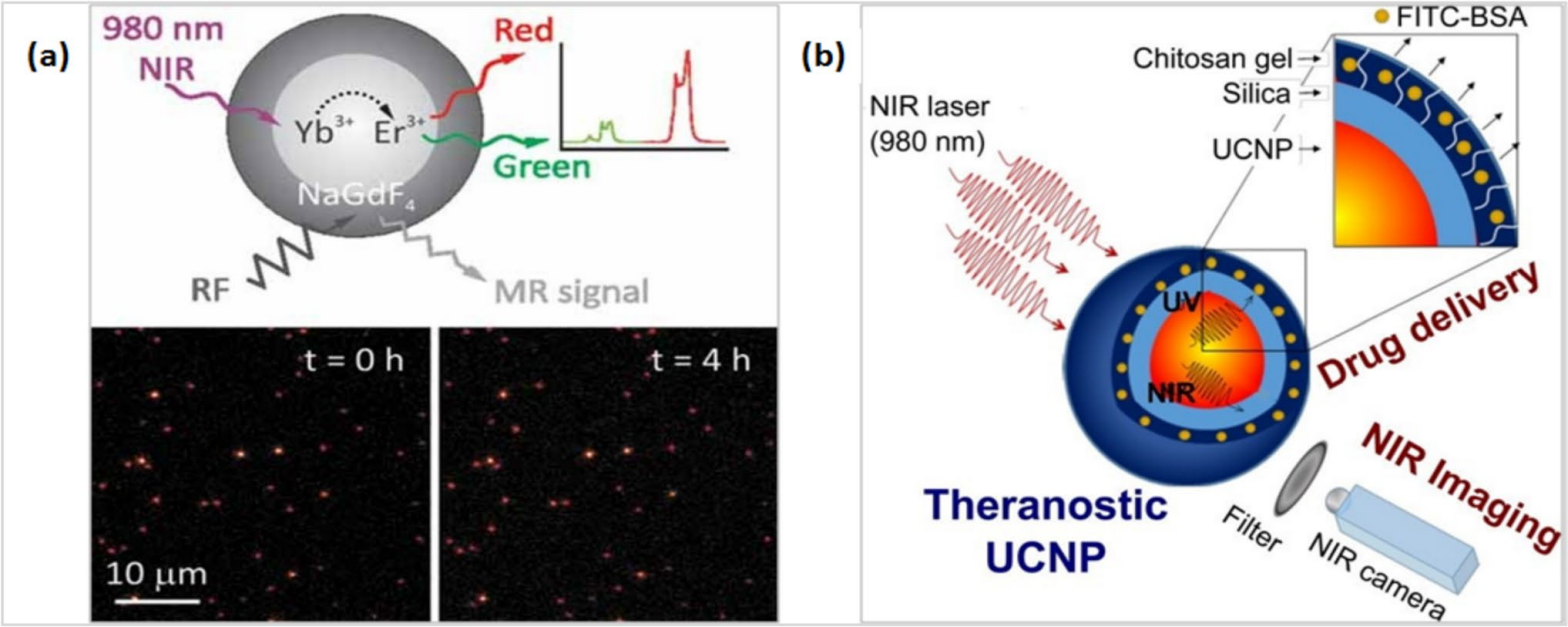
**a** Bright spots of Yb^3+^/Er^3+^ doped NaYF_4_ phosphors, (**b**) Upconversion effect of SiO_2-_NaGdF_4_:Nd^3+^,Yb.^3+^ composites under NIR imaging [130–132]

Upconversion nanoparticles (UCNPs) are also applied in the field of bioimaging due to proper characteristics of photostability, wide range absorptivity, brightness, and controlled continuous emission. These features make the upconverters suitable for working in the NIR region at orders of power magnitude lower than those for common investigation probes [5]. Recently, UNCPs with controlled sizes were developed thanks to the synthetic efforts that produce very fine nanoparticles more attuned to the many fields of bioimaging [8]. Lanthanide-doped UCNPs are synthesized now with highly adjusted phase, size, and dopant level [9]. These adjustable synthetic UCNPs release constant, bright luminescence with variable, selectable, and sensitive wavelengths making them suitable for the recent luminescence microscopy fields [5]. Yb^3+^/Er^3+^ doped NaYF_4_ is reported as the most common UCNPs used as bulk and nanocrystals since it properly reveals brightness during dark field imaging, as displayed in Fig. 22a [8]. The UC NaGdF_4_:Yb^3+^, Er^3+^ carried on NaGdF_4_:Nd^3+^, Yb^3+^ and combined with SiO_2_, was applied as a bioimaging of deep tissues since it gives a better response for excitation and emission in the IR and NIR regions as explained in Fig. 22b [130–132].

### Medical Therapy

**Fig. 23:**
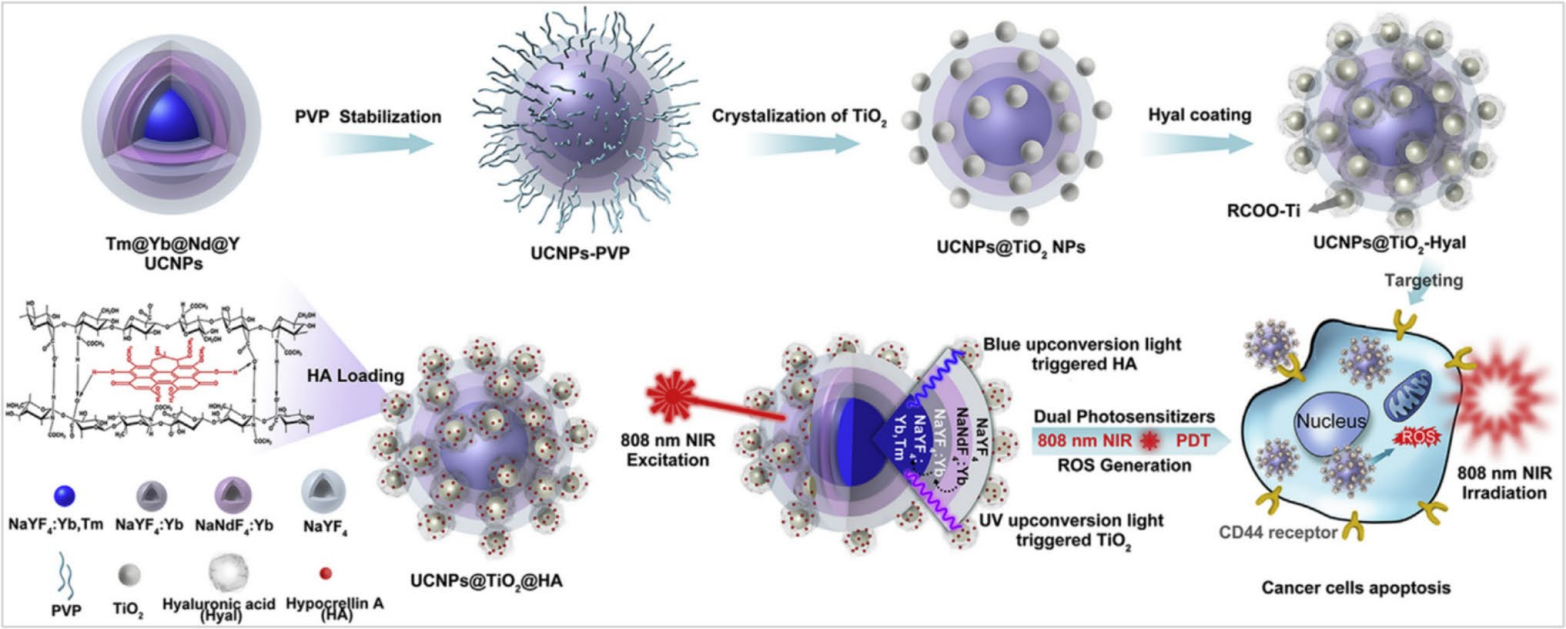
Illustration of 808 nm laser-induced dual-agent photosensitizing nanoplatforms by combining UV-blue UC emitting multi-shell UCNPs with TiO_2_ (UV-light-excited PS) and HA (blue-light-excited PS) [134]

One of the most common examples for medical thereby is employing the UCNPs in photodynamic therapy (PDT) 10 [133]. NIR-induced PDT works through the enhancement of the depth of tissue penetration to resonate the photosensitizers far from the upper layers [134]. This technique is still facing the overheating influence on the normal tissue cells, restricted tumor selectivity, and low yields of reactive oxygen species [134]. The UCNPs YF_4_:Yb/Tm@NaYF_4_, Yb@NaNdF_4_, and Yb@NaYF_4_ were developed as multishell nanostructures combined with TiO_2_ and hypocrellin A (HPA), which induce the apoptosis cancer cells by proper excitation-emission process illustrated in Fig. 23 [134]. YF_4_:Yb/Tm@NaYF_4_ core–shell structure combines the efficient upconversion properties of Yb/Tm-doped YF_4_ core with the enhanced biocompatibility and stability of NaYF_4_ shell. Yb@NaNdF_4_ and Yb@NaYF_4_ single-doped UCNPs offer simpler designs but might have lower upconversion efficiency compared to the core–shell structure. The additional TiO_2_ layer serves two purposes: 1) it acts as a photosensitizer, generating reactive oxygen species (ROS) upon UV irradiation, and 2) it enhances the light absorption and energy transfer to the UCNPs for improved upconversion efficiency. Finally, HPA photosensitizing drug works synergistically with ROS generated by TiO_2_, inducing apoptosis in cancer cells through singlet oxygen production.

### Optogenetics

**Fig. 24:**
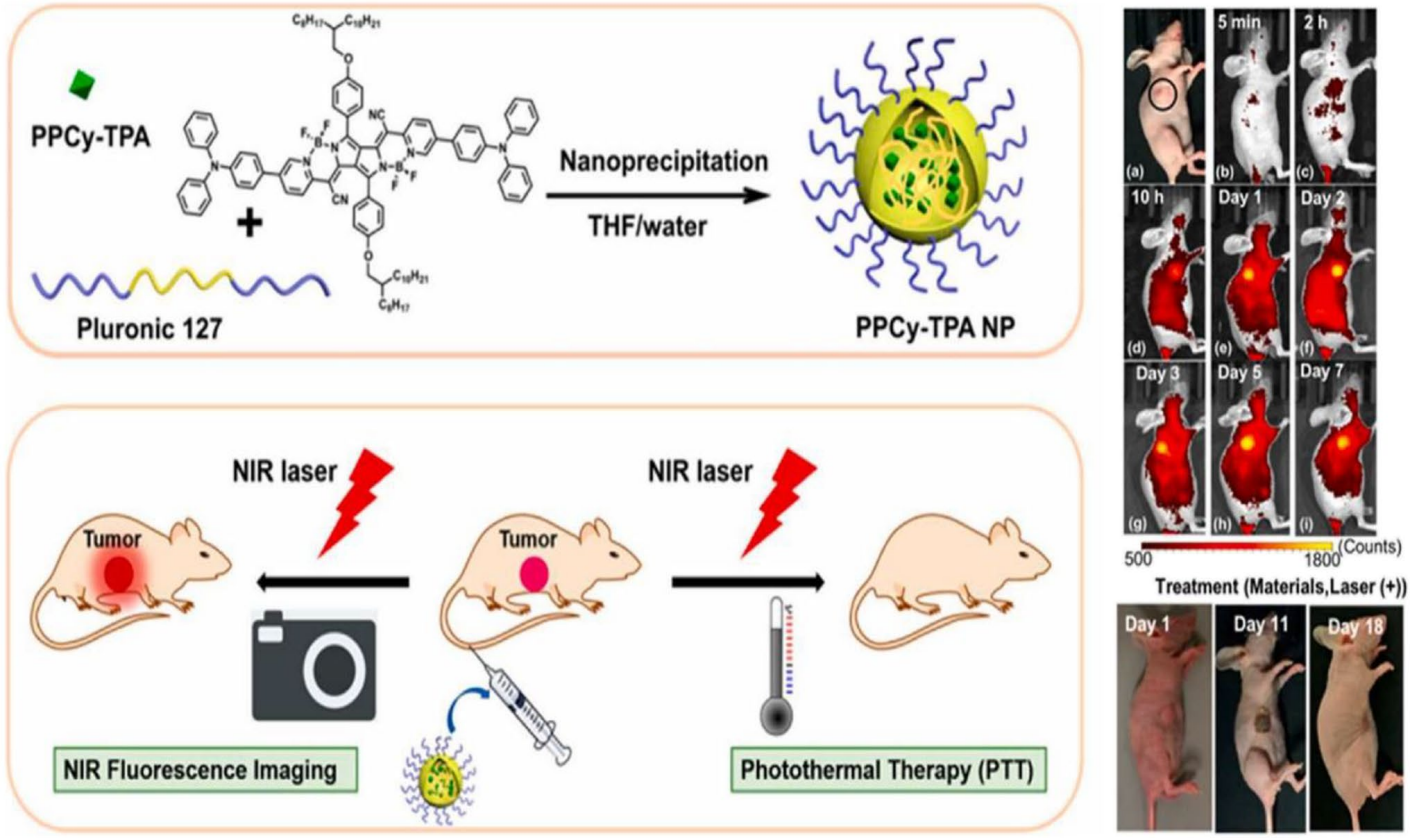
Encapsulation of polypyrrole cyanine (PPCy) derivatives within Pluronic F127 micelles, via nanoprecipitation, for the production of deep penetrating, and therapeutic NIR fluorescent probes for in-vivo imaging and photothermal ablation of tumors [138]

One of the most recent advanced applications of UCNPs is the optogenetic. Neurons activity and functions are controlled through optogenetic methods [135]. A definite wavelength of light spectrum is used to excite the neuron cells through specific biological processes, which act to activate or silence the neuronal activity. Two different biomedical uses of UCNPs were developed [136]. The first depended on the dye-synthesized core/active shell UCNP embedded poly(methyl methacrylate) polymer. The second utilizes the water-soluble UCNPs with Pluronic F127 with high upconversion performance, which were applied effectively in imaging in mouse models, as shown in Fig. 24 [135]. Optogenetics has been developed for the experimental interrogation of neuronal circuits and build a promise for the treatment of neurological troubles [137]. UCNPs act to absorb the light energy at the tissue penetrating the NIR region and release this energy in the visible light range helping to motivate the intense brain neurons [137, 138].

## Challenges and Future Prospects

Despite the promising potential of UC luminescent materials, several challenges remain to be addressed for their widespread application in PV solar cells and bioimaging.

### Challenges in PV Applications

The overall conversion efficiency of UC materials from low-energy to high-energy light is often limited, leading to lower-performing solar cells compared to traditional silicon designs. These materials can absorb low-energy photons (typically in the NIR region of the solar spectrum) and convert them into higher-energy photons (visible or UV). This ability allows them to utilize a broader range of the solar spectrum compared to silicon, potentially boosting solar cell efficiency. Not all absorbed low-energy photons get converted to high-energy ones. Some energy can be lost as heat or through other non-radiative processes. Excited states within the material can lose their energy through collisions with other molecules or vibrations, reducing the number of photons emitted. Emitted high-energy photons can be reabsorbed within the material itself, decreasing the usable light available for electricity generation. The specific wavelengths of high-energy photons emitted by the UC material might not perfectly match the absorption range of the underlying solar cell, leading to energy loss. Some UC materials, particularly lanthanide-based ones, can suffer from photodegradation and chemical instability, hindering their long-term performance in solar cells. During operation, solar cells are exposed to intense sunlight, which can excite electrons within the UC material. Some of these excited electrons can interact with the material's structure, causing defects and chemical changes. These defects act as trapping centres for other excited electrons, preventing them from participating in the UC process and leading to a decrease in efficiency over time. The extent of photodegradation depends on various factors like the lanthanide dopant type, host material composition, and processing conditions. Therefore, although UC materials offer the potential to expand the usable solar spectrum, their current practical efficiency in converting low-energy photons to high-energy ones for solar cell applications is often lower than ideal. This results in overall solar cell performance that falls short of the high efficiencies achieved by traditional silicon designs.

### Challenges in Biological Applications

Certain UC materials may exhibit toxicity towards living cells, raising concerns about their biocompatibility for in vivo imaging applications. This means that the materials can cause harm to cells, potentially including damage to membranes, proteins, or DNA. This harm can affect cell function and even lead to cell death. Biocompatibility refers to a material's ability to interact with living tissue without causing harmful effects. The potential toxicity of some UC materials raises concerns about their suitability for use in living organisms, particularly for imaging applications where they would be introduced into the body. During the UC process, some materials can generate free radicals and other reactive oxygen species that can damage cells. If the materials are not properly stabilized, certain ions (e.g., lanthanide ions) could leach out and disrupt cellular processes. Depending on their size and shape, UC materials could mechanically damage cells or block important biological pathways. While NIR excitation offers deeper tissue penetration advantages, many UC materials struggle with efficient luminescence in this region, restricting their imaging depth. Designing UC probes that specifically target biological targets with high affinity and minimal background interaction remains a challenge.

### Future Prospectives

The limitations of PV systems could be avoided through several processes such as material engineering, process optimization, and theoretical advancements are crucial for unlocking the full potential of UC materials solar cells applications. On the other hand, combining UC imaging with other modalities like MRI or CT scan is desirable for comprehensive medical diagnosis, although efficient integration and data interpretation present technical hurdles.

## Conclusion

This review discusses the fundamentals and mechanisms of UC processes, the structure, properties, and preparation methods of upconverter materials, as well as the applications of UC materials. Upconversion nanophosphors have been developed to be applied in various fields such as photocatalysis, biomedical, biosensing, bioimaging, optogenetics, drug release, and photodynamic therapy. The UC properties of luminescent materials can be affected by the host material, doping elements, and surface features. Accordingly, four main strategies are deeply discussed in this review to enhance the UC efficiency of these materials through (a) active core/inert shell structure, (b) core/active shell structures, (c) coupling with plasmonic structures, and (d) sensitization using synthetic dyes. Lanthanide-based UC materials can suffer from photodegradation and chemical instability, hindering their long-term performance in solar cells. In addition, some UC materials could cause harmful side effects in living organisms during bioimaging through emerging free radicals and other reactive oxygen species that could damage the organs' cells. Designing UC probes that specifically get over biological targets with high affinity and minimal background interaction remains a challenge. Extensive research on material engineering, process optimization, and theoretical advancements are needed, which considered crucial for unlocking the full potential of UC materials for PVs and biological applications.

## Data Availability

No datasets were generated or analysed during the current study.

## Abbreviations

UC: Upconversion
PV: Photovoltaic
PVs: Photovoltaics
TPV: Thermo Photovoltaic
UCNPs: Upconersion Nano Particles
Ln: Lanthanide
RE: Rare Earth
NIR: Near-Infrared
UV: Ultraviolet
ETU: Energy Transfer Upconversion
CUC: Cooperative Upconversion
PA: Photon Avalanche
TTA: Triplet-Triplet Annihilation
EMUC: Energy Migration-Mediated Upconversion
UCL: Upconversion Luminescence
ESA: Excited State Absorption
ET: Energy Transfer
CR ET: Cross Relaxation Energy Transfer
S_0_: Ground State
S_1_: Singlet State
T_1_: Triplet State
ISC: Intersystem Crossing
OLEDs: Organic Light-Emitting Devices
XRD: X-Ray Diffraction
DSSCs: Dye-Sensitized Solar Cells
QDSSCs: Quantum Dot-Synthesized Solar Cells
OSCs: Organic Solar Cells
SSFE: Selective Surfaces Front Emittance
SSBE: Selective Surfaces Back Emittance
RET: Resonance Energy Transfer
HPA: Hypocrellin A
ROS: Reactive Oxygen Species
PPCy: Polypyrrole Cyanine

## References

[1] Huang X (2017) Broadband dye-sensitized upconversion: A promising new platform for future Solar Upconverter Design. J Alloys Compd 690:356–359. 10.1016/j.jallcom.2016.08.142

[2] Dong H et al (2015) Efficient tailoring of upconversion selectivity by engineering local structure of lanthanides in NaxReF₃₊ₓ nanocrystals. J Am Chem Soc 137(20):6569–6576. 10.1021/jacs.5b01718 25938687 10.1021/jacs.5b01718

[3] Boriskina SV, Chen G (2014) Exceeding the solar cell Shockley-Queisser limit via thermal up-conversion of low-energy photons. Opt Commun 314:71–78. 10.1016/j.optcom.2013.10.042

[4] De Wild J et al (2010) Towards upconversion for amorphous silicon solar cells. Sol Energy Mater Sol Cells 94(11):1919–1922. 10.1016/j.solmat.2010.06.006

[5] Wen S et al (2019) Future and challenges for hybrid upconversion nanosystems. Nat Photonics 13(12):828–838. 10.1038/s41566-019-0528-x

[6] Peltomaa R et al (2021) Biosensing based on upconversion nanoparticles for food quality and safety applications. Analyst 146(1):13–32. 10.1039/d0an01883j 33205784 10.1039/d0an01883j

[7] Su Q et al (2016) Resonance energy transfer in upconversion nanoplatforms for selective biodetection. Acc Chem Res 50(1):32–40. 10.1021/acs.accounts.6b00382 27983801 10.1021/acs.accounts.6b00382

[8] Gargas DJ et al (2014) Engineering bright sub-10-nm upconverting nanocrystals for single-molecule imaging. Nat Nanotechnol 9(4):300–305. 10.1038/nnano.2014.29 24633523 10.1038/nnano.2014.29

[9] Liu Y et al (2017) Amplified stimulated emission in upconversion nanoparticles for super-resolution nanoscopy. Nature 543(7644):229–233. 10.1038/nature21366 28225761 10.1038/nature21366

[10] Chen G et al (2014) Upconversion nanoparticles: Design, nanochemistry, and applications in theranostics. Chem Rev 114(10):5161–5214. 10.1021/cr400425h 24605868 10.1021/cr400425h PMC4039352

[11] Bettinelli M, Carlos L, Liu X (2015) Lanthanide-doped upconversion nanoparticles. Phys Today 68(9):38–44. 10.1063/pt.3.2913

[12] Chen J, Zhao JX (2012) Upconversion nanomaterials: Synthesis, mechanism, and applications in sensing. Sensors 12(3):2414–2435. 10.3390/s120302414 22736958 10.3390/s120302414 PMC3376553

[13] Zhang H et al (2011) Composition tuning the upconversion emission in NaYF_4_:YB/TM hexaplate nanocrystals. Nanoscale 3(3):963. 10.1039/c0nr00823k 21264435 10.1039/c0nr00823k PMC3236245

[14] Yang J et al (2010) Mesoporous silica encapsulating upconversion luminescence rare-earth fluoride nanorods for secondary excitation. Langmuir 26(11):8850–8856. 10.1021/la904596x 20121245 10.1021/la904596x

[15] Zhou J et al (2010) Dual-modality in vivo imaging using rare-earth nanocrystals with near-infrared to near-infrared (NIR-to-NIR) upconversion luminescence and magnetic resonance properties. Biomaterials 31(12):3287–3295. 10.1016/j.biomaterials.2010.01.040 20132982 10.1016/j.biomaterials.2010.01.040

[16] Egger P et al (1996) Ba₂ErCl₇ ˗ A new near IR to near UV upconversion material. Adv Mater 8(8):668–672. 10.1002/adma.19960080816

[17] Maurya SK et al (2021) Assessment of colloidal NaGdF₄:Er^3^⁺/Yb^3^⁺ upconversion phosphor as contrast enhancer for optical coherence tomography. J Alloys Compd 865:158737. 10.1016/j.jallcom.2021.158737

[18] Mushtaq A, Yang X, Gao J (2022) Unveiling room temperature upconversion photoluminescence in monolayer WSe₂. Opt Express 30(25):45212. 10.1364/oe.471027 36522928 10.1364/OE.471027

[19] Granite S. (2023) Photon upconversion: Triplet triplet annihilation: Excited state absorption, Edinburgh Instruments. Available at: https://www.edinst.com/us/blog/photon-upconversion (Accessed: 09 May 2024).

[20] Goldschmidt JC, Fischer S (2015) Upconversion for photovoltaics – a review of materials, devices and concepts for performance enhancement. Adv Opt Mater 3(4):510–535. 10.1002/adom.201500024

[21] Joubert MF (1999) Photon avalanche upconversion in rare earth laser materials. Opt Mater 11(2–3):181–203. 10.1016/s0925-3467(98)00043-3

[22] Agazzi L, Wörhoff K, Pollnau M (2013) Energy-transfer-upconversion models, their applicability and breakdown in the presence of spectroscopically distinct ion classes: A case study in amorphous Al₂O₃:Er^3^⁺. J Phys Chem C 117(13):6759–6776. 10.1021/jp4011839

[23] Bednarkiewicz A, Szalkowski M (2022) Photon avalanche goes multicolour. Nat Nanotechnol 17(5):440–442. 10.1038/s41565-022-01100-9 35488077 10.1038/s41565-022-01100-9

[24] Ronchi A, Monguzzi A (2022) Sensitized triplet–triplet annihilation based photon upconversion in full organic and hybrid, multicomponent systems. Chem Phys Rev 3:041301. 10.1063/5.0112032

[25] Carrod AJ, Gray V, Börjesson K (2022) Recent advances in triplet–triplet annihilation upconversion and singlet fission, towards solar energy applications. Energy Environ Sci 15(12):4982–5016. 10.1039/d2ee01600a

[26] Bartusik-Aebisher D et al (2022) Photon upconversion in small molecules. Molecules 27(18):5874. 10.3390/molecules27185874 36144609 10.3390/molecules27185874 PMC9502815

[27] Seo SE et al (2022) Recent advances in materials for and applications of triplet–triplet annihilation-based upconversion. J Mater Chem C 10(12):4483–4496. 10.1039/d1tc03551g

[28] Yao B et al (2022) Recent advances in the photoreactions triggered by porphyrin-based triplet–triplet annihilation upconversion systems: Molecular innovations and nanoarchitectonics. Int J Mol Sci 23(14):8041. 10.3390/ijms23148041 35887385 10.3390/ijms23148041 PMC9323209

[29] Jiang H, Tao P, Wong WY (2023) Recent advances in triplet–triplet annihilation-based materials and their applications in electroluminescence. ACS Mater Lett 5(3):822–845. 10.1021/acsmaterialslett.2c01070

[30] Jiang Z et al (2013) Red-light-controllable liquid-crystal soft actuators via low-power excited upconversion based on triplet–triplet annihilation. J Am Chem Soc 135(44):16446–16453. 10.1021/ja406020r 24088066 10.1021/ja406020r

[31] Lin W et al (2023) Recent advances in triplet–triplet annihilation upconversion for bioimaging and biosensing. J Anal Test 7(4):327–344. 10.1007/s41664-023-00264-0

[32] Liu Q et al (2018) Highly photostable near-IR-excitation upconversion nanocapsules based on triplet–triplet annihilation for in vivo bioimaging application. ACS Appl Mater Interfaces 10(12):9883–9888. 10.1021/acsami.7b17929 29425018 10.1021/acsami.7b17929

[33] Wohnhaas C et al (2013) Triplet–triplet annihilation upconversion based nanocapsules for bioimaging under excitation by red and deep-red light. Macromol Biosci 13(10):1422–1430. 10.1002/mabi.201300149 23868857 10.1002/mabi.201300149

[34] Wang HQ et al (2012) Up-conversion semiconducting MoO₃:Yb/Er nanocomposites as buffer layer in organic solar cells. Sol Energy Mater Sol Cells 105:196–201. 10.1016/j.solmat.2012.06.005

[35] Song N, et al. (2019) Understanding the role of Yb^3^⁺ in the Nd/Yb coupled 808-nm-responsive upconversion. Front Chem 6. 10.3389/fchem.2018.00673. 10.3389/fchem.2018.00673 PMC6355672 30740392

[36] Ding M et al (2015) Li⁺ ions doping core–shell nanostructures: An approach to significantly enhance upconversion luminescence of lanthanide-doped nanocrystals. J Alloys Compd 623:42–48. 10.1016/j.jallcom.2014.10.089

[37] Balabhadra S et al (2021) Influence of the synthesis method on preferential clustering of Yb^3^⁺ in CaF₂:Yb^3^⁺/Er^3^⁺ upconverting nanoparticles. Opt Mater 112:110736. 10.1016/j.optmat.2020.110736

[38] Li Y et al (2020) Doping lanthanide nanocrystals with non-lanthanide ions to simultaneously enhance up- and down-conversion luminescence. Front Chem 8:832. 10.3389/fchem.2020.00832 33173764 10.3389/fchem.2020.00832 PMC7538674

[39] Chen X et al (2017) Fabrication and spectroscopic properties of Yb/Er:YAg and Yb, Er:YAg transparent ceramics by co-precipitation synthesis route. J Luminesc 188:533–540. 10.1016/j.jlumin.2017.05.008

[40] Zampiva RY et al (2018) Tunable green/red luminescence by infrared upconversion in biocompatible forsterite nanoparticles with high erbium doping uptake. Opt Mater 76:407–415. 10.1016/j.optmat.2018.01.004

[41] Chen YC, Chen TM (2011) Improvement of conversion efficiency of silicon solar cells using up-conversion molybdate La₂Mo₂O₉:Yb, R (R=Er, Ho) phosphors. J Rare Earths 29(8):723–726. 10.1016/s1002-0721(10)60530-3

[42] Wang X et al (2004) Luminescence spectroscopy and visible upconversion properties of Er^3^⁺ in ZnO nanocrystals. J Phys Chem B 108(48):18408–18413. 10.1021/jp048021t

[43] Peng S et al (2022) Upconversion luminescence and temperature sensing properties of Er^3^⁺/Yb^3^⁺ doped double-perovskite Ba₂LaNbO₆ phosphor. J Luminesc 242:118569. 10.1016/j.jlumin.2021.118569

[44] Sun J et al (2023) Upconversion luminescence of La₄Ti₉O₂₄: Er–Yb phosphor with high green color purity. Opt Mater 138:113656. 10.1016/j.optmat.2023.113656

[45] Shuai P et al (2022) Structure, optical characteristics and temperature sensing performance studies of Cs₃YF₆: Er^3^⁺, Yb^3^⁺ up-conversion material with cryolite structure. J Solid State Chem 306:122720. 10.1016/j.jssc.2021.122720

[46] Fu L, Wu Y, Fu T (2022) Determination of absorption cross-section of Re^3^⁺ in upconversion powder materials: Application to β-NaYF₄: Er^3^⁺. J Luminesc 245:118758. 10.1016/j.jlumin.2022.118758

[47] Yalan B, Yunfei Z (2020) Upconversion luminescence and optical thermometry of Pr^3^⁺-doped KLu₂F₇ phosphor. J Mater Sci Mater Electron 31(10):7991–7997. 10.1007/s10854-020-03339-1

[48] Cheng X et al (2019) Upconversion luminescence and optical temperature-sensing properties of LaNbO₄:Yb^3^⁺/Er^3^⁺ phosphors. J Electron Mater 49(1):518–523. 10.1007/s11664-019-07776-5

[49] Hernández-Rodríguez MA et al (2019) Upconversion and luminescence temperature sensitivity of Er^3^⁺ ions in yttrium oxysulfate nanophosphors. Opt Mater 95:109197. 10.1016/j.optmat.2019.109197

[50] Kang X et al (2019) Multicolor-tunable up-conversion emissions of Yb^3^⁺, E^r^3⁺/H^o^3⁺ co-doped Ba₃Lu₂Zn₅O₁₁: Crystal structure, luminescence and energy transfer properties. Dalton Trans 48(9):2917–2925. 10.1039/c8dt04577a 30644931 10.1039/c8dt04577a

[51] Roy A et al (2020) Enhanced multimodal behaviour of Tm^3^⁺/Yb^3^⁺ co-doped YTaO₄ ceramic material through Bi^3^⁺ activation and sensitization: Application as a spectral converter. Ceram Int 46(16):24893–24905. 10.1016/j.ceramint.2020.06.274

[52] Do Nascimento JP et al (2020) Up-conversion luminescence of Er^3^⁺/Pr^3^⁺/Yb^3^⁺ co-doped LaNbO₄ phosphors. J Electron Mater 49(10):6009–6015. 10.1007/s11664-020-08329-x

[53] Grigoroscuta M et al (2018) Enhanced near-infrared response of a silicon solar cell by using an up-conversion phosphor film of Yb/Er–co-doped CeO₂. Sol Energy 171:40–46. 10.1016/j.solener.2018.06.057

[54] Liao J et al (2019) Tunable upconversion luminescence and optical temperature sensing based on non-thermal coupled levels of Lu₃NbO₇:Yb^3^⁺/Ho^3^⁺ phosphors. Opt Mater 98:109452. 10.1016/j.optmat.2019.109452

[55] Li G et al (2018) Preparation and up-conversion luminescence of Yb^3^⁺/Er^3^⁺/GZO ceramics. Pol J Chem Technol 20(3):15–19. 10.2478/pjct-2018-0033

[56] Li X et al (2014) Energy transfer between Er3⁺ and Pr3⁺ for 2.7 μm fiber laser material. Fibers 2(1):24–33. 10.3390/fib2010024

[57] Lu W, Zhang J, Shi J (2019) Upconversion luminescence of Sr₈MgY(PO₄)₇:Yb^3^⁺–Er^3^⁺/Ho^3^⁺ phosphors for optical thermometry. J Mater Sci Mater Electron 30(19):17780–17786. 10.1007/s10854-019-02129-8

[58] Roy A et al (2021) Generation of color tunable emissions from Ho^3^⁺/Tm^3^⁺/Yb^3^⁺ co-doped YTaO₄ phosphors through NIR excitation under different conditions (variation of concentration, excitation pump power and the external temperature). J Alloys Compd 865:158938. 10.1016/j.jallcom.2021.158938

[59] Rivera VA et al (2012) Efficient plasmonic coupling between Er^3+^:(Ag/Au) in tellurite glasses. J Non-Cryst Solids 358(2):399–405. 10.1016/j.jnoncrysol.2011.10.008

[60] Aarts L et al (2011) Downconversion for the Er^3^⁺, Yb^3^⁺ couple in KPb₂Cl₅ - A low-phonon frequency host. J Lumin 131(4):608–613. 10.1016/j.jlumin.2010.10.041

[61] Kostka P et al (2021) Luminescence, up-conversion and temperature sensing in ER-doped TeO₂-PbCl₂-WO₃ glasses. J Non-Cryst Solids 553:120287. 10.1016/j.jnoncrysol.2020.120287

[62] Ming C, Song F, Ren X (2013) Color variety of up-conversion emission of Er^3^⁺/Yb^3^⁺ co-doped phosphate glass ceramics. Curr Appl Phys 13(2):351–354. 10.1016/j.cap.2012.08.011

[63] Taherunnisa S et al (2019) Effect of up-conversion luminescence in Er^3^⁺ doped phosphate glasses for developing erbium-doped fibre amplifiers (EDFA) and G-led’s. Opt Mater X 3:100034. 10.1016/j.omx.2019.100034

[64] Balaji S, Mandal AK, Annapurna K (2012) Energy transfer based NIR to visible upconversion: Enhanced red luminescence from Yb^3^⁺/Ho^3^⁺ co-doped tellurite glass. Opt Mater 34(11):1930–1934. 10.1016/j.optmat.2012.05.037

[65] Dousti MR et al (2012) Up-conversion enhancement in Er^3^⁺-Ag^1^⁺ co-doped zinc tellurite glass: Effect of heat treatment. J Non-Cryst Solids 358(22):2939–2942. 10.1016/j.jnoncrysol.2012.06.024

[66] Xin F et al (2012) Up-conversion luminescence of Er^3^⁺-doped glass ceramics containing β-NaGdF₄ nanocrystals for silicon solar cells. Mater Lett 78:75–77. 10.1016/j.matlet.2012.03.037

[67] Gopi D, Kanimozhi K, Kavitha L (2015) Opuntia ficus indica peel derived pectin mediated hydroxyapatite nanoparticles: Synthesis, spectral characterization, biological and antimicrobial activities. Spectrochim Acta A Mol Biomol Spectrosc 139:67–73 10.1016/j.saa.2015.01.039 25668694

[68] Kumar K, Rai SB, Rai DK (2007) Enhancement of luminescence properties in Er^3^⁺ doped TeO₂–Na₂O–PbX (X=O and F) ternary glasses. Spectrochim Acta Part A Mol Biomol Spectrosc 66(4–5):1052–1057. 10.1016/j.saa.2006.04.039 10.1016/j.saa.2006.04.039 16872889

[69] Cai W, Zhang Z, Jin Y, Lv Y, Wang L, Chen K, Zhou X (2019) Application of TiO₂ hollow microspheres incorporated with up-conversion NaYF₄:Yb^3^⁺, Er^3^⁺ nanoparticles and commercially available carbon counter electrodes in dye-sensitized solar cells. Sol Energy 188:441–449

[70] Li YC et al (2013) Effect of lanthanide doping on crystal phase and near-infrared to near-infrared upconversion emission of Tm^3^⁺ doped KF–YbF₃ nanocrystals. Ceram Int 39(7):7415–7424

[71] Zhang J et al (2012) Synthesis of NaYF₄:Yb/Er/Gd up-conversion luminescent nanoparticles and luminescence resonance energy transfer-based protein detection. Anal Biochem 421(2):673–679 22155069 10.1016/j.ab.2011.11.008 PMC3366261

[72] Sun C et al (2020) Solvothermal synthesis of lanthanide-doped NaYF₄ upconversion crystals with size and shape control: Particle properties and growth mechanism. ChemNanoMat 7(2):174–183

[73] Khan LU et al (2019) Synthesis and characterization of tunable color upconversion luminescence β-NaGdF₄:Yb^3^⁺, E^r^3⁺ nanoparticles. J Mater Sci Mater Electron 30(18):16856–16863

[74] Zhang H et al (2019) Titanium mesh-supported “TiO₂ nanowire arrays/Yb-Er-F tri-doped TiO₂ up-conversion nanoparticles” composite structure: Designation for high efficient flexible dye-sensitized solar cells. Thin Solid Films 681:103–113

[75] Jin J et al (2014) Upconversion luminescence of Ba(MoO₄)H(WO₄)₁₋ₓ:Yb^3^⁺/Er^3^⁺ nanocrystals synthesized through hydrothermal method. Opt Mater 37:371–375

[76] Yu J et al (2013) Er^3^⁺ and Yb^3^⁺ co-doped TiO₂−f up-conversion luminescence powder as a light scattering layer with enhanced performance in dye sensitized solar cells. J Power Sources 243:436–443

[77] Przybylska D et al (2019) Upconverting SrF₂ nanoparticles doped with Yb^3^⁺/Ho^3^⁺, Yb^3^⁺/Er^3^⁺ and Yb^3^⁺/Tm^3^⁺ ions – optimisation of synthesis method, structural, spectroscopic and cytotoxicity studies. Sci Rep 9(1):8669 31209230 10.1038/s41598-019-45025-1 PMC6572793

[78] Maurya SK et al (2021) Assessment of colloidal NaGdF₄:Er^3^⁺/Yb^3^⁺ upconversion phosphor as contrast enhancer for optical coherence tomography. J Alloys Compd 865:158737

[79] Song L et al (2017) Synthesis and up-conversion properties of Ho^3^⁺-Yb^3^⁺-F⁻ tri-doped TiO₂ nanoparticles and their application in dye-sensitized solar cells. Mater Res Bull 88:1–8

[80] Ma Z et al (2019) Yb^3^⁺/Er^3^⁺ co-doped Lu₂TeO₆ nanophosphors: Hydrothermal synthesis, upconversion luminescence and highly sensitive temperature sensing performance. J Alloys Compd 772:525–531

[81] Liang X et al (2017) Synthesis of hollow and mesoporous structured NaYF₄:Yb, Er upconversion luminescent nanoparticles for targeted drug delivery. J Rare Earths 35(5):419–429

[82] Xue B, Sun J (2013) Synthesis and tuning orange to green up-conversion color in Y₆WO₁₂:Er/Yb phosphor. Opt Mater 36(2):278–282

[83] Perrella RV et al (2014) Er^3^⁺-doped Y₂O₃ obtained by polymeric precursor: Synthesis, structure and upconversion emission properties. J Lumin 149:333–340

[84] Bedyal AK et al (2019) Excitation wavelength and Eu^3^⁺/Tb^3^⁺ content ratio dependent tunable photoluminescence from NaSrBO₃:Eu^3^⁺/Tb^3^⁺ phosphor. J Mater Sci Mater Electron 30(12):11714–11726

[85] Singh V et al (2013) NIR to visible frequency upconversion in Er^3^⁺ and Yb^3^⁺ co-doped BaZrO₃ phosphor. Spectrochim Acta A Mol Biomol Spectrosc 108:141–145 23466324 10.1016/j.saa.2013.01.073

[86] Fang D et al (2019) Application of bidirectional (up and down)-conversion luminescence material (GdBO₃:Yb^3^⁺/Tb^3^⁺) in CdSe₀.₄S₀.₆ quantum dot-sensitized solar cells. Opt Mater 88:80–90

[87] Li D et al (2015) Tailoring solar energy spectrum for efficient organic/inorganic hybrid solar cells by up-conversion luminescence nanophosphors. Electrochim Acta 182:416–423

[88] Liu W et al (2019) Upconversion luminescence and favorable temperature sensing performance of EULYTITE-type Sr₃Y(PO₄)₃:Yb^3^⁺/Ln^3^⁺ phosphors (Ln=Ho, Er, Tm). Sci Technol Adv Mater 20(1):949–963 31595178 10.1080/14686996.2019.1659090 PMC6764385

[89] Zheng J et al (2012) Intense 974 nm emission from ErₓYb₂₋ₓSi₂O₇ films through efficient energy transfer up-conversion from Er^3^⁺ to Yb^3^⁺ for Si solar cell. J Lumin 132(9):2341–2344

[90] Ming C et al (2012) Research on up- and down-conversion emissions of Er^3^⁺/Yb^3^⁺ co-doped phosphate glass ceramic. Opt Mater 35(2):244–247

[91] Deng M, Wang L (2014) Unexpected luminescence enhancement of upconverting nanocrystals by cation exchange with well retained small particle size. Nano Res 7(5):782–793

[92] Li C et al (2007) Different microstructures of β-NaYF₄ fabricated by hydrothermal process: Effects of pH values and fluoride sources. Chem Mater 19(20):4933–4942

[93] Li C, Quan Z et al (2017) Highly uniform and monodisperse β-NaYF₄:Ln^3^⁺ (Ln = Eu, Tb, Yb/Er, and Yb/Tm) hexagonal microprism crystals: Hydrothermal synthesis and luminescent properties. Inorg Chem 46(16):6329–6337 10.1021/ic070335i 17602610

[94] Mai H-X et al (2007) Size- and phase-controlled synthesis of monodisperse NaYF₄:Yb, Er nanocrystals from a unique delayed nucleation pathway monitored with upconversion spectroscopy. J Phys Chem C 111(37):13730–13739

[95] Mai H-X et al (2006) High-quality sodium rare-earth fluoride nanocrystals: Controlled synthesis and optical properties. J Am Chem Soc 128(19):6426–6436 16683808 10.1021/ja060212h

[96] Sun Q-C et al (2017) Photon upconversion towards applications in energy conversion and bioimaging. Prog Surf Sci 92(4):281–316

[97] Yang D et al (2018) A novel phosphor of Eu^3^⁺-activated Na₃GaF₆: Synthesis, structure, and luminescence properties. J Lumin 203:391–395

[98] Yang D et al (2020) Crystal structure and up-conversion luminescence properties of K₃ScF₆:Cr^3^⁺, Y^b^3⁺ cryolite. J Alloys Compd 848:156336

[99] Shuai P et al (2020) Preparation, structure and up-conversion luminescence properties of novel cryolite K₃YF₆:Er3⁺, Yb3⁺. RSC Adv 10(3):1658–1665 35494668 10.1039/c9ra10257d PMC9047056

[100] Zhou Y et al (2021) Abnormal thermally enhanced upconversion luminescence of lanthanide-doped phosphors: Proposed mechanisms and potential applications. J Mater Chem C 9(7):2220–2230

[101] Song N et al (2021) Enhancing upconversion of Nd^3^⁺ through Yb^3^⁺-mediated energy cycling towards temperature sensing. J Rare Earths 39(12):1506–1511

[102] Wu DM et al (2014) Plasmon-enhanced upconversion. J Phys Chem Lett 5(22):4020–4031 26276488 10.1021/jz5019042

[103] Meijer MS et al (2018) Absolute upconversion quantum yields of blue-emitting LiYF₄:Yb^3^⁺, T^m^3⁺ upconverting nanoparticles. Phys Chem Chem Phys 20(35):22556–22562 30155527 10.1039/c8cp03935f

[104] Zhou B et al (2015) Controlling upconversion nanocrystals for emerging applications. Nat Nanotechnol 10(11):924–936. 10.1038/nnano.2015.251 26530022 10.1038/nnano.2015.251

[105] Lu K et al (2019) Temperature-independent lifetime and thermometer operated in a biological window of upconverting NaErF₄ nanocrystals. Nanomaterials 10(1):24 31861808 10.3390/nano10010024 PMC7022627

[106] Wisser MD et al (2018) Improving quantum yield of upconverting nanoparticles in aqueous media via emission sensitization. Nano Lett 18(4):2689–2695 29589449 10.1021/acs.nanolett.8b00634

[107] Tian Q, Tao K, Sun K (2013) β-NaYF₄:Yb, Er at β-NaYF₄ core/shell nanocrystals with significantly enhanced upconversion fluorescence by a successive two-step hot-injection approach. Micro Nano Lett 8(10):731–734

[108] Dong C et al (2012) Cation exchange: A facile method to make NaYF₄:Yb, Tm-NaGdF₄ core–shell nanoparticles with a thin, tunable, and uniform shell. Chem Mater 24(7):1297–1305

[109] Vetrone F et al (2009) The active-core/active-shell approach: A strategy to enhance the upconversion luminescence in lanthanide-doped nanoparticles. Adv Funct Mater 19(18):2924–2929

[110] Ding X et al (2017) Multifunctional core/satellite polydopamine@Nd^3^⁺-sensitized upconversion nanocomposite: A single 808 nm near-infrared light-triggered theranostic platform for in vivo imaging-guided photothermal therapy. Nano Res 10(10):3434–3446

[111] Zhuo Z et al (2017) Manipulating energy transfer in lanthanide-doped single nanoparticles for highly enhanced upconverting luminescence. Chem Sci 8(7):5050–5056 29568476 10.1039/c7sc01393k PMC5846170

[112] Xiao H et al (2021) Core–shell structured upconversion/lead-free perovskite nanoparticles for anticounterfeiting applications. Angew Chem Int Ed 61(8):e202115136 10.1002/anie.202115136 34918447

[113] Xie X et al (2013) Mechanistic investigation of photon upconversion in Nd^3^⁺-sensitized core–shell nanoparticles. J Am Chem Soc 135(34):12608–12611 23947580 10.1021/ja4075002

[114] Meng Y et al (2022) Bright single-nanocrystal upconversion at sub 0.5 W CM⁻2 irradiance via coupling to single nanocavity mode. Nat Photon 17(1):73–81

[115] Zhang H et al (2010) Plasmonic modulation of the upconversion fluorescence in NaYF₄:Yb/Tm hexaplate nanocrystals using gold nanoparticles or nanoshells. Angew Chem Int Ed 49(16):2865–2868 10.1002/anie.200905805 PMC3027842 20235253

[116] Chen G et al (2015) Energy-cascaded upconversion in an organic dye-sensitized core/shell fluoride nanocrystal. Nano Lett 15(11):7400–7407 26487489 10.1021/acs.nanolett.5b02830 PMC4915588

[117] Gao J et al (2020) Near-infrared light-induced self-powered aptasensing platform for aflatoxin B1 based on upconversion nanoparticles-doped Bi₂S₃ nanorods. Anal Chem 93(2):677–682 33284587 10.1021/acs.analchem.0c04248

[118] Zhang S et al (2022) A synergistic effect of NaYF₄:Yb, Er@NaGdF₄:Nd@SiO₂ upconversion nanoparticles and TiO₂ hollow spheres to enhance photovoltaic performance of dye-sensitized solar cells. Electrochim Acta 421:140435

[119] Schulze TF, Schmidt TW (2015) Photochemical upconversion: Present status and prospects for its application to solar energy conversion. Energy Environ Sci 8(1):103–125. 10.1039/c4ee02481h

[120] Chen W et al (2015) Sub-bandgap photon harvesting for organic solar cells via integrating up-conversion nanophosphors. Org Electron 19:113–119. 10.1016/j.orgel.2015.01.036

[121] Zhang Z et al (2018) Near-infrared-plasmonic energy upconversion in a nonmetallic heterostructure for efficient H2 evolution from ammonia borane. Adv Sci 5(9):1800748. 10.1002/advs.201800748 10.1002/advs.201800748 PMC6145233 30250807

[122] Wang J et al (2020) Synergistic effects of lanthanide surface adhesion and photon-upconversion for enhanced near-infrared responsive photodegradation of organic contaminants in wastewater. Environ Sci: Nano 7(11):3333–3342. 10.1039/d0en00670j

[123] Yang W et al (2022) Yb^3^⁺, Er^3^⁺: CeF₃ crystals: Growth, first-principles simulation, and near-infrared optical properties. J Luminescence 252:119257. 10.1016/j.jlumin.2022.119257

[124] Batista V et al. (2021) Introducing special issue on photocatalysis and Photoelectrochemistry. J Chem Phys 154(19). 10.1063/5.0053681 10.1063/5.0053681 34240913

[125] Wu Y et al (2021) Multiphoton upconversion materials for photocatalysis and environmental remediation. Chem - Asian J 16(18):2596–2609. 10.1002/asia.202100751 34403201 10.1002/asia.202100751

[126] Wei J et al (2022) Upconversion boosting pollutants degradation efficiency in wide-spectrum responsive photocatalysts. Chemosphere 309:136679. 10.1016/j.chemosphere.2022.136679 36195128 10.1016/j.chemosphere.2022.136679

[127] Zhang Y et al (2019) Single near-infrared-laser driven Z-scheme photocatalytic H_2_ evolution on upconversion material@Ag3PO4@black phosphorus. Chem Eng J 375:121967. 10.1016/j.cej.2019.121967

[128] Liu X et al (2018) A novel “modularized” optical sensor for pH monitoring in biological matrixes. Biosens Bioelectron 109:150–155. 10.1016/j.bios.2018.02.052 29550738 10.1016/j.bios.2018.02.052

[129] Gu B et al (2016) Thiazole derivative-modified upconversion nanoparticles for Hg^2+^ detection in living cells. Nanoscale 8(1):276–282. 10.1039/c5nr05286f 26607020 10.1039/c5nr05286f

[130] Xu J et al (2017) Highly emissive dye-sensitized upconversion nanostructure for dual-photosensitizer photodynamic therapy and bioimaging. ACS Nano 11(4):4133–4144. 10.1021/acsnano.7b00944 28320205 10.1021/acsnano.7b00944

[131] Chen C et al (2018) Multi-photon near-infrared emission saturation nanoscopy using upconversion nanoparticles. Nat Commun 9(1):3290. 10.1038/s41467-018-05842-w 30120242 10.1038/s41467-018-05842-w PMC6098146

[132] Li X et al (2013) Nd^3^⁺ sensitized up/down converting dual-mode nanomaterials for efficient in-vitro and in-vivo bioimaging excited at 800 nm. Sci Rep 3(1):3536. 10.1038/srep03536 24346622 10.1038/srep03536 PMC3866591

[133] Patel M et al (2022) Recent development in upconversion nanoparticles and their application in optogenetics: A Review. J Rare Earths 40(6):847–861. 10.1016/j.jre.2021.10.003

[134] Hou Z et al (2016) 808 nm light-triggered and hyaluronic acid-targeted dual-photosensitizers nanoplatform by fully utilizing Nd^3^⁺-sensitized upconversion emission with enhanced anti-tumor efficacy. Biomaterials 101:32–46. 10.1016/j.biomaterials.2016.05.024 27267626 10.1016/j.biomaterials.2016.05.024

[135] Mahmoudi P, Veladi H, Pakdel F (2017) Optogenetics, tools and applications in Neurobiology. J Med Signals Sens 7(2):71. 10.4103/2228-7477.205506 28553579 PMC5437765

[136] Mahata MK, De R, Lee KT (2021) Near-infrared-triggered upconverting nanoparticles for biomedicine applications. Biomedicines 9(7):756. 10.3390/biomedicines9070756 34210059 10.3390/biomedicines9070756 PMC8301434

[137] Wu X et al (2016) Dye-sensitized core/active shell upconversion nanoparticles for optogenetics and bioimaging applications. ACS Nano 10:1060–1066. 10.1021/acsnano.5b06383 26736013 10.1021/acsnano.5b06383 PMC4913696

[138] Chen S, Liu X, McHugh T (2019) Near-infrared deep brain stimulation via upconversion nanoparticle-mediated optogenetics. Opt Biopsy XVII: Toward Real-Time Spectrosc Imaging Diagnosis 5:550. 10.1117/12.2506055

